# Supplementary material for: Examining the acceptability of actigraphic devices in children using qualitative and quantitative approaches: protocol for a systematic review and meta-analysis
Source: BMJ Open. 2023 Mar 1;13(3):e070597. doi: 10.1136/bmjopen-2022-070597 (PMC9980313; doi:10.1136/bmjopen-2022-070597)
Supplement: Supplementary data [file bmjopen-2022-070597supp002.pdf]

EMBASE via OVID

1. child*.ab,ti.
2. exp child/
3. primary school.ab,ti.
4. youth*.ab,ti.
5. kindergar#en.ab,ti.
6. kid*.ab,ti.
7. pupil*.ab,ti.
8. juvenile*.ab,ti.
9. exp juvenile/
10. young people*.ab,ti.
11. 1 or 2 or 3 or 4 or 5 or 6 or 7 or 8 or 9 or 10
12. (actigraph* or actimet* or actograp* or actomet* or acceleromet*).ab,ti.
13. motor activity.ab,ti.
14. exp motor activity/
15. Fitbit.ab,ti.
16. ((electronic or remote or wearable or fitness or activity) adj3 (track* or monitor* or wearable* or device* or technolo*)).ab ,ti.
17. step count*.ab,ti.
18. 12 or 13 or 14 or 15 or 16 or 17
19. acceptability.ab,ti.
20. experience*.ab,ti.
21. perception*.ab,ti.
22. feasibility.ab,ti.
23. feedback.ab,ti.
24. design*.ab,ti.
25. usability.ab,ti.
26. practicability.ab,ti.
27. willingness.ab,ti.
28. usefulness.ab,ti.
29. engagement.ab,ti.
30. opinion*.ab,ti.
31. 19 or 20 or 21 or 22 or 23 or 24 or 25 or 26 or 27 or 28 or 29 or 30
32. 11 and 18 and 31
