# Supplementary material for: Examining the acceptability of actigraphic devices in children using qualitative and quantitative approaches: protocol for a systematic review and meta-analysis
Source: BMJ Open. 2023 Mar 1;13(3):e070597. doi: 10.1136/bmjopen-2022-070597 (PMC9980313; doi:10.1136/bmjopen-2022-070597)
Supplement: Supplementary data [file bmjopen-2022-070597supp004.pdf]

PsychINFO via OVID

1. child*.ab,ti.
2. primary school.ab,ti.
3. youth*.ab,ti.
4. kindergar#en.ab,ti.
5. kid*.ab,ti.
6. pupil*.ab,ti.
7. juvenile*.ab,ti.
8. young people*.ab,ti.
9. 1 or 2 or 3 or 4 or 5 or 6 or 7 or 8
10. (actigraph* or actimet* or actograp* or actomet* or acceleromet*).ab,ti.
11. motor activity.ab,ti.
12. Fitbit.ab,ti.
13. ((electronic or remote or wearable or fitness or activity) adj3 (track* or monitor* or wearable* or device* or technolo*)).ab ,ti.
14. step count*.ab,ti.
15. 10 or 11 or 12 or 13 or 14
16. acceptability.ab,ti.
17. experience*.ab,ti.
18. perception*.ab,ti.
19. feasibility.ab,ti.
20. feedback.ab,ti.
21. design*.ab,ti.
22. usability.ab,ti.
23. willingness.ab,ti.
24. usefulness.ab,ti.
25. engagement.ab,ti.
26. opinion*.ab,ti.
27. 16 or 17 or 18 or 19 or 20 or 21 or 22 or 23 or 24 or 25 or 26
28. 9 and 15 and 27
