# Supplementary material for: Examining the acceptability of actigraphic devices in children using qualitative and quantitative approaches: protocol for a systematic review and meta-analysis
Source: BMJ Open. 2023 Mar 1;13(3):e070597. doi: 10.1136/bmjopen-2022-070597 (PMC9980313; doi:10.1136/bmjopen-2022-070597)
Supplement: Supplementary data [file bmjopen-2022-070597supp005.pdf]

## Social Policy and practice via OVID

1. child*.ab,ti.
2. primary school.ab,ti.
3. youth*.ab,ti.
4. kid*.ab,ti.
5. pupil*.ab,ti.
6. juvenile*.ab,ti.
7. young people*.ab,ti.
8. 1 or 2 or 3 or 4 or 5 or 6 or 7
9. (actigraph* or actimet* or actograp* or actomet* or acceleromet*).ab,ti.
10. ((electronic or remote or wearable or fitness or activity) adj3 (track* or monitor* or wearable* or device* or technolo*)).ab ,ti.
11. 9 or 10
12. acceptability.ab,ti.
13. experience*.ab,ti.
14. perception*.ab,ti.
15. feasibility.ab,ti.
16. feedback.ab,ti.
17. design*.ab,ti.
18. usability.ab,ti.
19. willingness.ab,ti.
20. usefulness.ab,ti.
21. engagement.ab,ti.
22. opinion*.ab,ti.
23. 12 or 13 or 14 or 15 or 16 or 17 or 18 or 19 or 20 or 21 or 22
24. 8 and 11 and 23
