# Supplementary material for: Examining the acceptability of actigraphic devices in children using qualitative and quantitative approaches: protocol for a systematic review and meta-analysis
Source: BMJ Open. 2023 Mar 1;13(3):e070597. doi: 10.1136/bmjopen-2022-070597 (PMC9980313; doi:10.1136/bmjopen-2022-070597)
Supplement: Supplementary data [file bmjopen-2022-070597supp006.pdf]

ERIC via EBSCOhost

S1 - TI child* OR AB child* OR TI "primary school" OR AB "primary school" OR TI youth* OR AB youth* OR TI kindergar?en OR AB kindergar?en OR TI kid* OR AB kid* OR TI pupil* OR AB pupil* OR TI juvenile* OR AB juvenile* OR TI "young people*" OR AB "young people*"

S2 - TI ((actigraph* or actimet* or actograp* or actomet* or acceleromet*)) OR AB ((actigraph* or actimet* or actograp* or actomet* or acceleromet*)) OR TI "motor activity" OR AB "motor activity" OR TI fitbit OR AB fitbit OR TI (((electronic or remote or wearable or fitness or activity) adj3 (track* or monitor* or wearable* or device* or technolo*))) OR AB (((electronic or remote or wearable or fitness or activity) adj3 (track* or monitor* or wearable* or device* or technolo*))) OR TI "step count*" OR AB "step count*"

S3 - TI acceptability OR AB acceptability OR TI experience* OR AB experience* OR TI perception* OR AB perception* OR TI feasibility OR AB feasibility OR TI feedback OR AB feedback OR TI design* OR AB design* OR TI usability OR AB usability OR TI practicability OR AB practicability OR TI willingness OR AB willingness OR TI usefulness OR AB usefulness OR TI engagement OR AB engagement OR TI opinion* OR AB opinion*

S4 – S1 AND S2 AND S3
