# Supplementary material for: Examining the acceptability of actigraphic devices in children using qualitative and quantitative approaches: protocol for a systematic review and meta-analysis
Source: BMJ Open. 2023 Mar 1;13(3):e070597. doi: 10.1136/bmjopen-2022-070597 (PMC9980313; doi:10.1136/bmjopen-2022-070597)
Supplement: Supplementary data [file bmjopen-2022-070597supp009.pdf]

Full data extraction form

Data Extraction Form		Review date:
		Ref ID:
Author, year, date		
Title		
Journal/source		
Publication type, status		
Location of study		
Qualitative/quantitative		
Aims		
Design		
Number of participants		Drop out:
Information on age		
Information on sex		
Diagnosis		
Inclusion/exclusion criteria		
Device name, description (inc. mode of delivery)		
Device purpose		
Device wear location		
Protocol wear time and intensity		Actual:
Information on protocol adherence		
Information on device engagement/incentivisation		
Acceptability assessment measures		
Description of Outcomes		
Notes		
