# Supplementary material for: Examining the acceptability of actigraphic devices in children using qualitative and quantitative approaches: protocol for a systematic review and meta-analysis
Source: BMJ Open. 2023 Mar 1;13(3):e070597. doi: 10.1136/bmjopen-2022-070597 (PMC9980313; doi:10.1136/bmjopen-2022-070597)
Supplement: Author's manuscript [file bmjopen-2022-070597.draft_revisions.pdf]

BMJ Open

BMJ Open is committed to open peer review. As part of this commitment we make the peer review history of every article we publish publicly available.

When an article is published we post the peer reviewers' comments and the authors' responses online. We also post the versions of the paper that were used during peer review. These are the versions that the peer review comments apply to.

The versions of the paper that follow are the versions that were submitted during the peer review process. They are not the versions of record or the final published versions. They should not be cited or distributed as the published version of this manuscript.

BMJ Open is an open access journal and the full, final, typeset and author-corrected version of record of the manuscript is available on our site with no access controls, subscription charges or pay-per-view fees (http://bmjopen.bmj.com).

If you have any questions on BMJ Open's open peer review process please email
info.bmjopen@bmj.com

BMJ Open

Examining the acceptability of actigraphic devices in children using qualitative and quantitative approaches: protocol for a systematic review and meta-analysis

Journal:	*BMJ Open*
Manuscript ID	bmjopen-2022-070597
Article Type:	Protocol
Date Submitted by the Author:	05-Dec-2022
Complete List of Authors:	Morris, Anna; King's College London Institute of Psychiatry Psychology and Neuroscience, Child and Adolescent Psychiatry<br>Telesia, Laurence; King's College London Institute of Psychiatry Psychology and Neuroscience, ; South London and Maudsley Mental Health NHS Trust,<br>Wickersham, Alice; King's College London, Institute of Psychiatry, Psychology and Neuroscience;<br>Epstein, Sophie; South London and Maudsley NHS Foundation Trust, NIHR Maudsley Biomedical Research Centre<br>Matcham, Faith; University of Sussex, School of Psychology<br>Sonuga-Barke, Edmund; King's College London,<br>Downs, Johnny; Kings College London, Institute of Psychiatry, Psychology and Neuroscience
Keywords:	Child & adolescent psychiatry < PSYCHIATRY, Health informatics < BIOTECHNOLOGY & BIOINFORMATICS, Community child health < PAEDIATRICS

SCHOLARONE™
Manuscripts

Examining the acceptability of actigraphic devices in children using qualitative and quantitative approaches: protocol for a systematic review and meta-analysis

Morris, A. C[1]., Telesia, L[1,2]., Wickersham, A [3,4]., Epstein, S[3]., Matcham, F. A. E[5]., Sonuga-Barke, E. J. S[1]., & Downs, J. M. (2021)[1,2].

[1] King's College London, London, UK, Department of Child and Adolescent Psychiatry, Institute of Psychiatry Psychology and Neuroscience; [2] South London and Maudsley NHS Foundation Trust, London, UK; [3] King's College London, London, UK, Department of Psychological Medicine, Institute of Psychiatry Psychology and Neuroscience & NIHR South London and Maudsley Biomedical Research Centre;[4] NIHR Maudsley Biomedical Research Centre, South London and Maudsley NHS Foundation Trust, London, UK; [5] School of Psychology, University of Sussex, Falmer, UK

- anna.morris@kcl.ac.uk
- laurence.telesia@kcl.ac.uk
- alice.wickersham@kcl.ac.uk
- sophie.epstein@kcl.ac.uk
- f.matcham@sussex.ac.uk
- edmund.sonuga-barke@kcl.ac.uk
- johnny.downs@kcl.ac.uk

Correspondence: Anna Morris, Department of Child and Adolescent Psychiatry, Institute of Psychiatry, Psychology and Neuroscience, King's College London, PO Box 84, De Crespigny Park, London SE5 8AF, UK; Email: anna.morris@kcl.ac.uk

For peer review only

Abstract

Introduction: Actigraphy is commonly used to record free-living physical activity in both typically and atypically developing children. While the accuracy and reliability of actigraphy have been explored extensively, research regarding young people's opinion towards these devices is scarce. This review aims to identify and synthesise evidence relating to the acceptability of actigraphic devices in 5-11-year olds.

Methods and analysis: Database searches will be applied to EMBASE; MEDLINE and PsychINFO. Supplementary forward and backward citation and grey literature database

searches, including Healthcare Management Information Consortium (HMIC) and PsycEXTRA will be conducted. Qualitative and quantitative studies, excluding review articles and meta-analyses, will be eligible, without date restrictions. Article screening and data extraction will be undertaken by two review authors and disagreements will be deferred to a third reviewer. The primary outcome, actigraphic acceptability, will derive from the narrative synthesis of the main themes identified from included qualitative literature, and pooled descriptive statistics relating to acceptability identified from quantitative literature. Subgroup analyses will determine if acceptability changes as a function of the key participant and actigraphic device factors.

Ethics and dissemination: Ethical approval is not required for this systematic review as it uses data from previously published literature. The results will be presented in a manuscript and published in a peer-review journal and will be considered alongside a separate stream of co-design research to inform the development of a novel child-worn actigraphic device.

PROSPERO Registration number: CRD42021232466

Strengths and limitations of this study

- This systematic review and meta-analysis will be the first to synthesise literature exploring the acceptability of actigraphic devices in children, a topic where evidence is sparse.
- Detailed study methodology and statistical analysis strategy are planned, to enable the inclusion of quantitative and qualitative literature.
- Comprehensive search terms are informed by a theoretical framework to enhance the likelihood of detecting literature relevant to acceptability.
- Literature not written in English will be excluded and may lead pertinent studies to be omitted.
- Narrative synthesis of qualitative evidence may be influenced by reviewer subjectivity and must be critically appraised.

Background

Actigraphy, an objective measure of movement, is widely considered the preferred method for activity-based monitoring in clinical and epidemiological research. [1] Modern actigraphic devices, known as actigraphs are small, inexpensive, and readily accessible rendering them

For peer review only - http://bmjopen.bmj.com/site/about/guidelines.xhtml

ideal for inclusion in studies investigating free-living patterns of physical activity and sleep-wake cycles. [2] Actigraphs are credited for their ability to overcome important measurement biases associated with traditional subjective methods including memory, perception, and social desirability bias to produce a more detailed and accurate account of motor activity. [3] These benefits are particularly pronounced in the study of young children, where parent or teacher-reported questionnaires are often relied upon as the primary source of information, [4] and where self-reported measures tend to be inaccurate. [5, 6]

Actigraphs have been applied extensively in the remit of children's physical and mental health, especially in the context of sleep medicine, where actigraphic devices are used to record nocturnal movement patterns in children. [7] Actigraphic devices are increasingly relied upon to examine sleep patterns in childhood neurological disorders [8] and neurodevelopmental disorders. [9, 10] Deficiencies in sleep quantity or quality are also linked to the onset of various health difficulties, [11, 12] behavioural problems, and cognitive impairments. [13] Therefore, objective measures of sleep are increasingly used to support clinical assessment and management of sleep disturbances or disorders. [4] Compared to more costly data collection methods such as polysomnography which typically records sleep information for one or two nights in a laboratory, actigraphy can be used in naturalistic environments for extended periods, [14] making it easier to collect data from children who might find it difficult to sleep normally in unfamiliar surroundings. [15, 16]

Considerable efforts have also been made to deploy actigraphs to assess physical activity in children, including obesity, [17, 18] and chronic conditions such as cerebral palsy. [19] Particular attention is also being paid to the potential role of actigraphy in the assessment and diagnosis of Attention Deficit Hyperactivity Disorder (ADHD), [20] a common childhood disorder characterised by the persistent presence of three core symptoms, namely impulsivity, inattention, and hyperactivity. [21, 22] Within clinical practice, diagnosis of ADHD is usually based upon a clinical assessment of the individual, supplemented by collateral history and reports from parents/carers and teachers. [21, 23, 24, 25] However, the clinical utility of these measures has been questioned, as the main features of the disorder are rarely observed directly or systematically measured by members of the clinical decision-making team. [26] In contrast, actigraphy provides the means to objectively monitor physical activity, a well-established means of determining the severity of hyperactive symptoms, [27] across a variety of different contexts.

There is a considerable body of evidence focusing on the accuracy of actigraphic outcomes in the context of physical activity and sleep, [28, 29, 30] however, the question of acceptability

For peer review only - http://bmjopen.bmj.com/site/about/guidelines.xhtml

has often been overlooked. Engagement with medical devices is heavily dependent on aesthetic preferences [31] and is potentially even more pertinent for children, who may have unique physical feature requirements compared to adults. [32] Children with neurodevelopmental disorders may also experience particular tactile sensitivities associated with the actigraphic device or its placement, [33] and stigma associated with visible markers of medical investigation. [34] Therefore, it is essential to design actigraphs that incorporate the needs and preferences of children to ensure sustained engagement with these products, particularly when asking children to wear them continuously over prolonged periods in-home and school-based environments.

Aims

This review aims to examine the literature pertaining to the acceptability of body-worn actigraphic devices for children aged 5-11 years old. Whilst this review may discover overlap between features considered acceptable by both typically and atypically developing children, differences between these groups will be highlighted if identified. If available, we aim also to include feedback on acceptability from parents/caregivers of young children who may provide insightful feedback about the acceptability of these devices. As such, the results of this review may have several practical applications, both in typically and atypically developing children. For example, by offering information to clinical teams on the types of actigraphic devices that are likely to be well tolerated by children under their care or guiding the development of future study protocols to include child-accepted actigraphs.

Further, we aim to use the findings from this review alongside data gathered from concurrent co-design research, to inform the design of a bespoke actigraphic device primarily indented to monitor physical activity within this age group.

Methods and analysis

This protocol has been prospectively recorded in the PROSPERO international database. We used the Preferred Reporting Items for Systematic Review and Meta-Analysis protocols (PRISMA-P) checklist when writing our report [35]

Eligibility criteria

For peer review only - http://bmjopen.bmj.com/site/about/guidelines.xhtml

Population

We are principally interested in actigraphic acceptability among primary school-aged children. In the UK, this represents children aged 5-11, an age range that typically encompasses most primary school-aged children worldwide. Therefore, studies will be included in this review if they involve children (where at least 50% fall within the 5-11 year age range or where the average age falls within this age range) in the design, development, or evaluation of an actigraphic device. Additionally, parents and caregivers of young children often act as the 'gatekeeper' responsible for enabling or restricting access to or time spent interacting with wearable technologies, [36, 37] therefore, studies including parents or caregiver/s of children aged between 5-11 years old will also be included.

Exposure and outcome

Studies will be eligible for this review if they investigate the development or use of an actigraph and report a corresponding measure of acceptability. In this review acceptability will be defined according to the Theoretical Framework of Acceptability (TFA), [38] including participants' affective (e.g. feelings), cognitive (e.g. perceptions) or behavioural (e.g. attrition rates) response towards the actigraph. Included studies may use actigraphs for any purpose, as the primary focus of the study or as part of a multifaceted tool. Participants must be required to wear an actigraphic device in a community setting to be included in this review. Studies will be excluded if they required the wearable device to be worn in only a lab-based setting.

Study design

Qualitative studies, specifically co-design, focus groups and interview methodologies will be included as well as quantitative studies including feasibility, pilot, observational cohort, or intervention studies. Reviews, meta-analyses, and protocols will be excluded, as will commentaries, opinion pieces, editorials, magazine articles, and technical or internal reports. As patient-centred design and acceptability literature is an emerging field we will extend inclusion to published conference abstracts if they provide sufficient information to demonstrate that they meet the inclusion criteria. Likewise, letters that describe the methods and results of otherwise unpublished research may also be considered for inclusion. No date restrictions will be applied to this search. Studies can be published or unpublished but must be written in English. See Table 1 for more detailed inclusion and exclusion criteria.

For peer review only - http://bmjopen.bmj.com/site/about/guidelines.xhtml

Table 1. Inclusion/exclusion table for studies exploring actigraphic devices in children.

	Inclusion criteria	Exclusion criteria
Publication status	- Studies written in English<br>- Published or unpublished studies	- Studies written in other languages<br>- Commentaries, opinion pieces, editorials, magazine articles and technical or internal reports
Study design	- Qualitative or quantitative studies	- Reviews or meta-analyses
Device	- Studies in which a wearable activity tracker to measure motor activity was worn by a child within the target age group	- Studies which do not employ any wearable devices<br>- Studies using wearable devices which measure parameters other than motor activity (e.g., balance, number of steps, proportion of time sitting etc.)<br>- Studies in which wearable activity trackers were discussed, but not worn
Age	- Studies where all participants are aged between 5-11 years, as indicated by age range, or reported school year<br>- Studies reporting a measure of central tendency (e.g., median, mean) which falls within the range of 5-11 years<br>- Studies that present data in a way that allows extraction for a subgroup aged between 5-11 years<br>- Studies that satisfy the above age criteria for the child wearing the device, but feedback is provided by a caregiver or other relevant stakeholder	- Studies where all participants are outside the target age of 5-11 years<br>- Studies where it is not possible to extract data specific for participants aged between 5-11 years
Setting	- Studies with protocols which involves the wear of an activity tracker in a community setting, regardless of whether the device is also worn in a non-community setting	- Studies which take place entirely in a non-community setting (e.g., laboratory, university or hospital setting)
Acceptability	- Studies which present a qualitative or quantitative measure of acceptability<br>- Measures can include focus group or interview feedback, proportions of participants declining to wear the device or proportion and extent of deviation from the study's wear protocol	- Studies which do not contain any direct or indirect measure of acceptability

For peer review only - http://bmjopen.bmj.com/site/about/guidelines.xhtml

Information sources and search strategy

The electronic databases Embase, MEDLINE, PsychInfo and Social Policy and Practice through the OVID interface; and Education Resources Information Center (ERIC), British Education Index and CINAHL through the EBSCO interface will be searched using a combination of free text and thesaurus terms.

The search terms will derive from three key concepts, children (population), actigraphy (exposure) and acceptability (outcome). Search terms and advanced search techniques will be utilised, including truncation, wild card and adjacency operators, which will be tailored for each database as required. We will also review the reference list of included articles and related existing review articles (backward citation searching), and articles which have referenced included studies (forward citation searching). Both forward and backward citation searching will be conducted in Web of Science Core Collection, and if a citation is unavailable in this source, Google Scholar will be queried.

Additional grey literature searches will be conducted within PsycEXTRA, a complementary database to PsychINFO and the Healthcare Management Information Consortium (HMIC), comprising records from the Department of Health (DoH) and the King's Fund. Supplementary searches will be carried out using the same search strategy described above for the primary electronic databases. In addition, consultation with experts will be conducted and authors of studies included in the review will be contacted to inquire about any known studies matching the inclusion criteria. A preliminary search strategy for Embase is provided in online supplement 1.

Data management

The search output will be downloaded into Mendeley reference management software. Duplicates will automatically be detected and then removed by the reviewers. The remaining references will be exported to a Microsoft Excel document and manually screened for undetected duplicates.

Study selection process

Two reviewers (AM and LT) will each independently screen a 10% sample of the titles and abstracts generated by the search strategy. Interrater reliability for this sample will be

For peer review only - http://bmjopen.bmj.com/site/about/guidelines.xhtml

determined using Cohen’s kappa. [39] If the interrater reliability is considered “substantial” or “almost perfect”, based on a kappa statistic greater than 0.60 [40] each review author will independently review 50% of the remaining abstracts, with the option to jointly discuss unclear or otherwise challenging abstracts. If the strength of agreement for the 10% overlap sample is lower, both authors will review all remaining abstracts. Any disagreements between the reviewers regarding suitability for inclusion will be rectified by joint decision and, if the dispute continues, a third reviewer (JD) will decide. Full-text screening will be performed for studies deemed eligible or of unclear eligibility, again by the same two independent reviewers and a third reviewer where disagreement regarding inclusion persists. AM and LT will perform backward and forward citation searches to identify any other relevant literature. To establish agreement, this process will be conducted by both review authors (AM & LT) for 10% of the included reference lists, and the remainder evenly distributed between the authors for independent screening. Further study inclusion will be determined using the previously described consensus approach. All article exclusions will be recorded and will be justified for any articles removed at the full-text stage.

Data items and extraction process

Each paper selected for inclusion will be reviewed and data contained within the paper will be extracted and recorded using a data extraction form adapted from the Cochrane Review Group Data Extraction Form (online supplementary file 2). Both review authors (AM & LT) will perform data extraction and any disagreements regarding this procedure will be discussed and settled by a third reviewer (JD) if required. Before starting, extraction consistency will be examined by piloting the form with 10% of eligible studies. Any disagreements arising from this process will be resolved through discussion between review authors (AM and LT), and a third reviewer will decide on unresolved disagreements (JD). If needed, supplementary information will be requested directly from study authors to finalise inconsistencies and complete extraction.

For both qualitative and quantitative papers, extracted data will include - study characteristics (e.g. aims, design, sample size and analysis), information source (e.g. journal article or conference abstract), country and date of origin, title, and publication status. Participant descriptive data, including age, gender, and mental/physical health status will be recorded. Where provided, details regarding the actigraphic device will also be recorded, including device name, purpose (e.g. sleep, obesity, ADHD symptom tracking), features (e.g. material, design and functionality), body-worn location and mode of delivery (e.g. on its own or part of a multi-faceted tool).

For peer review only - http://bmjopen.bmj.com/site/about/guidelines.xhtml

All text relating to relevant qualitative findings will be collected from the results section of included papers, to record themes and sub-themes pertaining to child-worn actigraph acceptability and engagement. Particular attention will be paid to any design or contextual factors that hinder or promote actigraphic device use in the target population. Where available, this will include direct participant quotes to afford greater data granularity at the data synthesis stage. Additionally, descriptions of the research methods used to assess acceptability (e.g. structured questionnaire with space to report free text, structured or semi-structured interview or focus groups) and the information reporting source (e.g. child, caregiver, or teacher) will be collected.

Where provided, actigraph monitoring protocol adherence data will be extracted - that is the proportion of time in the given measurement period that the actigraphic device was actually worn for the duration specified in the study methods, according to self-report e.g. diary entry, or activity monitoring device data. We will also extract absolute numbers for the duration and intensity of prescribed and actual wear time, to investigate adherence according to protocol differences. Attrition rates and protocol deviations, that is the percentage of consented participants who stop wearing the study device or wore the device in a different way than directed within the observed timeframe, as well as any comments for this behaviour will be captured. Where studies also use questionnaires, scales, or other methods to ascertain acceptability/useability, the name and content of the method, as well as participants' responses will be recorded. Information on factors which could influence participant device engagement will also be extracted. This could include but is not limited to, frequency of contact with the research team, explicit reminders to wear the device, for example, phone calls from the research team or automated text or email reminders and incentivisation. As this review focuses on the acceptability rather than the effectiveness of actigraphs, data relating to changes in behavioural outcomes following the use of an actigraphic device (e.g. improved sleep or increased physical activity) will not be collected.

If a study is found which includes at least 50% of the children aged 5-11 years as well as a minority outside of this age group, we will aim to extract data related only to the age group of interest. If it is not possible to do so, this will be highlighted in the finalised report.

Quality assessment

The relevant Critical Appraisal Skills Programme (CASP) UK [41] checklists will be used to assess the quality of included articles according to the study design. Quality assessment for each paper included in the review will be independently conducted by two review authors (AM and LT), and any appraisal discrepancies will be resolved by a third reviewer (JD).

For peer review only - http://bmjopen.bmj.com/site/about/guidelines.xhtml

Studies will not be excluded based on methodological quality, as the primary aim of this review is to identify literature about end users' experiences interacting with actigraphs. Rather, the appraisal will be used to comment on the general quality of available literature regarding the acceptability of body-worn actigraphic devices in children.

Data synthesis and analysis

This review will utilise a systematic narrative synthesis approach. [42] The characteristics and key results for all included studies will first be presented in a table and elaborated on in the main body of the text. Summarised data will include, study aims, the types of actigraphic devices, details about the participant group and specific constructs of acceptability that were assessed.

For qualitative studies, the main themes, data collection method and analysis method (e.g. thematic, grounded theory or content analysis) will be presented. As outlined by Levitt [43], to meta-analyse the qualitative studies, data relevant to the review question will be selected from the included studies to form "meaning units" which will be compared with those from other studies to develop common themes and higher-order categories. Depending on the number and type of papers identified by the search, it may be more appropriate to simply summarise the findings at this stage. However, to allow meaningful comparisons, where possible the results will also be presented by subgroups, that is, according to device types or specific participant factors to better surmise any aesthetic requirements that are shared between or are unique to certain populations. Potential subgroups include, but will not be limited to, whether the children of interest are typically or atypically developing or have been diagnosed with a mental or physical health condition. Similarly, as the eligibility criteria span a period of considerable developmental change, the patterns of acceptability by age will be explored, as well as gender and ethnicity.

Descriptive statistics relating to acceptability will be summarised for quantitative studies, including percentages, frequencies, and measures of central tendency. If multiple studies are included that overlap sufficiently in methodology and outcome variables, random effects meta-analytical procedures will be used. For example, if possible, a pooled estimate of proportional wear time will be produced accompanied by a 95% confidence interval (CI) using the Stata command Metaprop. The same procedure will be applied where two or more studies employ the same acceptability questionnaires or other outcome measures. For example, if compliance data is presented as a binary outcome, such as a frequency count of participants deviating from prescribed wear protocol or proportion failing to achieve a minimum wear-time, the relative risk for that outcome will be calculated and meta-analytic

For peer review only - http://bmjopen.bmj.com/site/about/guidelines.xhtml

methods for dichotomous outcomes will be considered, Statistical heterogeneity will be reported using the I^2 statistic. If ten or more studies are identified for inclusion within a meta-analysis, techniques will be used to assess publication bias where appropriate, including Egger's regression test and funnel plots. Where possible, results stratified by actigraphic device type will be determined, and if the data permits, subgroup analyses will be reported according to the participant variables described for the qualitative subgroup analyses strategy.

Patient and public involvement

No patients were involved in the conception or development of this project.

Discussion

This systematic review seeks to identify and summarise the existing literature relating to actigraphic device acceptability in young children and highlight particular features that make actigraphic products more or less palatable in childhood. To our knowledge, this is the first synthesis to focus on child and parental acceptance of actigraph use in free-living settings for this age group, despite the potential impact of acceptability on wear time. [30, 32]

Our intention to include studies which focus on both the subjective, experiential reports from children and caregivers, as well as objective markers of wear time and attrition will allow a more comprehensive understanding of the role particular features within actigraphs play. For example, if qualitative reports consistently raise a feature as relevant, it may be possible to use a quantitative measure to corroborate this by comparing studies using devices with that feature to those without. Conversely, this approach may contradict the qualitative reports, thus identifying areas for further exploration and future research.

We hope that, by including studies with a range of prescribed wear times as well as focusing primarily on community settings, our findings will have an impact on real-world research and clinical applications. For example, some features may promote compliance in the short term only, whereas another feature is associated with better prolonged wear. This knowledge, along with an understanding of the preferences of specific subgroups, would allow clinicians and researchers to select the optimal device for their needs, and support designers to develop new products targeted to specific applications. This is timely given the rise of commercially available actigraphs purposefully developed for this age group. [36]

For peer review only - http://bmjopen.bmj.com/site/about/guidelines.xhtml

Furthermore, by collating and meta-analysing wear time and attrition data from several studies we hope to set a benchmark, against which new devices may be compared. Such a benchmark could be helpful to device designers, particularly those wishing to evaluate prototypes against existing models in an iterative design process.

To strengthen this review, we have used a theoretical framework, the TFA to develop the search strategy, as well as use broad search terms to maximise the chances of identifying papers relevant to the study question. A possible limitation is that the process of deducing themes across varied qualitative studies has been criticised owing to the potential interference of reviewer subjectivity on the study findings. [44] However, we have brought together a team of reviewers from multi-disciplinary backgrounds to minimise any undue influence from any one reviewer's judgment. Our decision to exclude non-English written literature may limit the number of papers identified but is necessary due to resource constraints.

Ethics and dissemination

This systematic review will use existing data and therefore does not require ethical approval. Findings from the review will be published in a peer-review journal and used to inform the development of a new actigraphic device.

Footnotes:

Contributors: AM (corresponding author) and LT coconceived and developed the review protocol, defined the search strategy, and drafted the initial, revised, and finalised manuscripts. AM and LT will perform data extraction and analysis and interpret the review findings. AW and SE provided critical revisions to the study design and manuscript. JD, ESB and FM co-conceived the review, supported the study design and amendments to the manuscript.

Funding statement:

This review is funded by the National Institute for Health Research (NIHR) Clinician Science Fellowship award (CS-2018-18-ST2-014). LT is funded by the NHIR as an Academic Clinical Fellow. AW is in receipt of a PhD studentship funded by the NIHR Biomedical Research Centre (BRC) at South London and Maudsley NHS Foundation Trust and Kings College London (NIHR-INF-0690). SE is funded by a Medical Research Council (MRC) Clinical Research Training Fellowship (MR/T001437/1) and previously received salary support from

For peer review only - http://bmjopen.bmj.com/site/about/guidelines.xhtml

an MQ Data Science Award and from the Psychiatry Research Trust. JD is funded by the Clinician Science Fellowship award (CS-2018-18-ST2-014) and received additional funding from an MRC Clinical Research Training Fellowship (MR/L017105/1) and Psychiatry Research Trust Peggy Pollak Research Fellowship in Developmental Psychiatry. ESB is funded by the BRC, MRC (MR/T046864/1), Economic and Social Research Council (ESRC; ES/S004467/1), UK Research and Innovation's (UKRI) ESRC (ES/V016393/1) and NIHR Programme Grants for Applied Research (PGfAR; RP-PG-0618-20003).

Disclaimer: This paper represents independent research [part] funded by the NIHR Biomedical Research Centre at South London and Maudsley NHS Foundation Trust and King's College London. The views expressed are those of the author(s) and not necessarily those of the NHS, the NIHR or the Department of Health and Social Care. This communication [part] reflects the views of the RADAR-CNS consortium and neither IMI nor the European Union and EFPIA are liable for any use that may be made of the information contained herein. The funding body has not been involved in the design of the study, the collection or analysis of data, or the interpretation of data.

Competing interests statement: None declared.

References:

1. Migueles, J. H., Cadenas-Sanchez, C., Ekelund, U., Delisle Nyström, C., Mora-Gonzalez, J., Löf, M., Labayen, I., Ruiz, J. R., & Ortega, F. B. (2017). Accelerometer data collection and processing criteria to assess physical activity and other outcomes: A systematic review and practical considerations. *Sports Medicine*, 47(9),1821-1845. doi: 10.1007/s40279-017-0716-0.

2. Martin, J. L., & Hakim, A. D. (2011). Wrist actigraphy. *Chest*, 139(6), 1514-1527. doi: 10.1378/chest.10-1872.

3. Arvidsson, D., Fridolfsson, J., & Borjesson, M. (2019). Measurement of physical activity in clinical practice using accelerometers. *Journal of Internal Medicine*, 286, 137–153. doi:10.1111/joim. 12908

4. Mazza, S., Bastuji, H., & Rey, A. E. (2020). Objective and subjective assessments of sleep in children: Comparison of actigraphy, sleep diary completed by children and parents' estimation. *Frontiers in psychiatry*, 11, 495. doi: 10.3389/fpsyt.2020.00495

For peer review only - http://bmjopen.bmj.com/site/about/guidelines.xhtml

5. Adamo, K. B., Prince, S. A., Tricco, A. C., Connor-Gorber, S., & Tremblay, M. A. (2009). Comparison of indirect versus direct measures for assessing physical activity in the pediatric population: A systematic review, *International Journal of Pediatric Obesity*, 4:1, 2-27. doi: 10.1080/17477160802315010

6. Chinapaw, M. J. M., Mokkink, L. B., van Poppel, M. N. M., van Mechelen, W., & Terwee, C. B. (2010). Physical activity questionnaires for youth: A systematic review of measurement properties. *Sports Medicine*, 40 (7), 539-563. doi:10.2165/11530770-000000000-00000.

7. Meltzer, L. J., Montgomery-Downs, H. E., Insana, S. P., & Walsh, C. M. (2012). Use of actigraphy for assessment in pediatric sleep research. *Sleep Medicine Reviews*, 16(5):463-75. doi: 10.1016/j.smrv.2011.10.002.

8. Tsai, S. Y., Lee, W. T., Jen, S. F., Lee, C., & Weng, W. C. (2018). Sleep and behavior problems in children with epilepsy. *Journal of Pediatric Health Care*, 18, 30123-8. doi: 10.1016/j.pedhc.2018.07.004

9. Merbler, A. M., Byiers, B. J., Garcia, J. J., Feyma, T. J., & Symons, F. J. (2018). The feasibility of using actigraphy to characterize sleep in Rett syndrome. *Journal of Neurodevelopmental Disorders*, 10(1), 8. doi: 10.1186/s11689-018-9227-z.

10. De Crescenzo, F., Licchelli, S., Ciabattini, M., Menghini, D., Armando, M., Alfieri, P., Mazzone, L., Pontrelli, G., Livadiotti, S., Foti, F., Quested, D., & Vicari, S. (2016). The use of actigraphy in the monitoring of sleep and activity in ADHD: A meta-analysis. *Sleep medicine reviews*, *26*, 9–20. doi: 10.1007/s12402-013-0122-x

11. Cappuccio, F. P., Taggart, F. M., Kandala, N. B., Currie, A., Peile, E., Stranges, S., & Miller, M. A. (2008). Meta-analysis of short sleep duration and obesity in children and adults. *Sleep*, 31, 619–26. doi:10.1093/sleep/31.5.619

12. Smaldone, A., Honig, J. C., & Byrne, M. W. (2007). Sleepless in America: Inadequate sleep and relationships to health and well-being of our nation's children. *Pediatrics*, 119, S29–37. doi:10.1542/peds.2006-2089F.

For peer review only - http://bmjopen.bmj.com/site/about/guidelines.xhtml

13. Maski, K. P., & Kothare, S. V. (2013). Sleep deprivation and neurobehavioral functioning in children. *International Journal of Psychophysiology*, 89, 259–64. doi: 10.1016/j.ijpsycho.2013.06.019.

14. Verhulst, S. L., Schrauwen, N., De Backer, W. A., & Desager, K. N. (2006). First night effect for polysomnographic data in children and adolescents with suspected sleep disordered breathing. *Archives of Disease in Childhood*, 91, 233–7. doi:10.1136/adc.2005.085365.

15. Sadeh, A., Alster, J., Urbach, D., & Lavie, P. (1989). Actigraphically based automatic bedtime sleep-wake scoring: Validity and clinical applications. *Journal of Ambulatory Monitoring*, 2, 209–216.

16. Sadeh, A., Lavie, P., Scher, A., Tirosh, E., & Epstein, R. (1991). Actigraphic home-monitoring sleep-disturbed and control infants and young children: A new method for pediatric assessment of sleep-wake patterns. *Pediatrics*, 87, 494–499.

17. Jiménez-Pavón, D., Kelly, J., & Reilly, J. J. (2010). Associations between objectively measured habitual physical activity and adiposity in children and adolescents: Systematic review. *International Journal of Pediatric Obesity*, 5(1), 3-18. doi: 10.3109/17477160903067601.

18. Wilks, D. C., Besson, H., Lindroos, A. K., Ekelund, U. (2011). Objectively measured physical activity and obesity prevention in children, adolescents and adults: A systematic review of prospective studies. *Obesity Reviews*, 12(5), e119-29. doi: 10.1111/j.1467-789X.2010.00775.x.

19. Keawutan, P., Bell, K., Davies, P. S., & Boyd, R. N. (2014). Systematic review of the relationship between habitual physical activity and motor capacity in children with cerebral palsy. *Research in Developmental Disabilities*, 35(6), 1301-9. doi: 10.1016/j.ridd.2014.03.028.

20. Subcommittee on Attention-Deficit/Hyperactivity Disorder, Steering Committee on Quality Improvement and Management, Wolraich, M., Brown, L., Brown, R. T., DuPaul, G., Earls, M., Feldman, H. M., Ganiats, T. G., Kaplanek, B., Meyer, B., Perrin, J., Pierce, K., Reiff, M., Stein, M. T., & Visser, S. (2011). ADHD: Clinical practice guideline for the diagnosis, evaluation, and treatment of attention-

For peer review only - http://bmjopen.bmj.com/site/about/guidelines.xhtml

deficit/hyperactivity disorder in children and adolescents. *Pediatrics*, *128*(5), 1007–1022. doi:10.1542/peds.2011-2654.

21. De Crescenzo, F., Armando, M., Mazzone, L., Ciliberto, M., Sciannamea, M., Figueroa, C., Janiri, L., Quested, D., & Vicari, S. (2014). The use of actigraphy in the monitoring of methylphenidate versus placebo in ADHD: A meta-analysis. *Attention deficit and hyperactivity disorders*, *6*(1), 49–58. doi: 10.1007/s12402-013-0122-x.

22. National Institute for Health & Care Excellence. (2018). *Attention Deficit Hyperactivity Disorder: Diagnosis and Management of ADHD in Children, Young People and Adults (National Clinical Practice Guideline Number 87)*. Retrieved from: https://www.nice.org.uk/guidance/ng87/resources/attention-deficit-hyperactivity-disorder-diagnosis-and-management-pdf-1837699732933

23. American Psychiatric Association. (2013). *Diagnostic and statistical manual of mental disorders (5th ed.).* Washington, D.C.: American Psychiatric Association

24. McConaughy, S. H., Ivanova, M., Antshel, K., Eiraldi, R. B., & Dumenci, L. (2009). Standardized observational assessment of Attention Deficit Hyperactivity Disorder combined and predominantly inattentive subtypes: II. Classroom observations. *School Psychology Review*, 39, 362–381.

25. Wolraich, M. L., Chan, E., Froehlich, T., Lynch, R. L., Bax, A., Redwine, S. T., Ihyembe, D., & Hagan, J. F. Jr. (2019). ADHD diagnosis and treatment guidelines: A historical perspective. *Pediatrics*, 144(4), e20191682. doi: 10.1542/peds.2019-1682.

26. Hall, C. L., Taylor, J. A., Newell, K., Baldwin, L., Sayal, K., & Hollis, C. (2016). The challenges of implementing ADHD clinical guidelines and research best evidence in routine clinical care settings: Delphi survey and mixed-methods study. *BJPsych Open*, 2(1), 25-31. doi: 10.1192/bjpo.bp.115.002386

27. Hall, C. L., Valentine, A. Z., Groom, M. J., Walker, G. M., Sayal, K., Daley, D., Hollis, C. (2016). The clinical utility of the continuous performance test and objective measures of activity for diagnosing and monitoring ADHD in children: A systematic review. *European Child and Adolescent Psychiatry*, 25(7), 677-99. doi: 10.1007/s00787-015-0798-x.

For peer review only - http://bmjopen.bmj.com/site/about/guidelines.xhtml

28. Kam, H. J., Lee, K., Cho, S. M., Shin, Y. M., Park, R. W. (2011). High-resolution actigraphic analysis of ADHD: A wide range of movement variability observation in three school courses - a pilot study. *Healthcare Informatics Research*, 17(1), 29-37. doi: 10.4258/hir.2011.17.1.29.

29. Evenson, K. R., Goto, M. M., & Furberg, R. D. (2015). Systematic review of the validity and reliability of consumer-wearable activity trackers. *The international journal of behavioral nutrition and physical activity*, 12, 159. doi: https://doi.org/10.1186/s12966-015-0314-1

30. Scott, H., Lack, L., & Lovato, N. (2020). A systematic review of the accuracy of sleep wearable devices for estimating sleep onset. *Sleep medicine reviews*, 49, 101227. doi: 10.1016/j.smrv.2019.101227.

31. O'Connor, S., Hanlon, P., O'Donnell, C. A., Garcia, S., Glanville, J., & Mair, F. S. (2016). Understanding factors affecting patient and public engagement and recruitment to digital health interventions: A systematic review of qualitative studies. *BMC Medical Information Decision Making*. 16(1), 120. doi: 10.1186/s12911-016-0359-3

32. Geljins, A. C., Killelea, B., Vitale, M., Mankad, V., & Moskowitz. A. (2006). Appendix C. The dynamics of pediatric device innovation: putting evidence in context. Safe Medical Devices for Children. Washington, DC: *The National Academies Press*. doi: 17226/11313

33. Fawkes, D. B., Malow, B. A., Weiss, S. K., Reynolds, A. M., Loh, A., Adkins, K. W., Wofford, D. D., Wyatt, A. D., Goldman, S. E. (2014). Conducting actigraphy research in children with neurodevelopmental disorders-a practical approach. *Behavioral Sleep Medicine*,12, 116. doi: 10.1080/15402002.2013.854245.

34. Mueller, A. K., Fuermaier, A. B., Koerts, J., & Tucha, L. (2012). Stigma in attention deficit hyperactivity disorder. *Attention Deficit Hyperactivity Disorder*. 4(3),101-14. doi: 10.1007/s12402-012-0085-3.

35. Moher, D., Shamseer, L., Clarke, M., Ghersi, D., Liberati, A., Petticrew, M., et al. (2015). Preferred reporting items for systematic review and meta-analysis protocols

For peer review only - http://bmjopen.bmj.com/site/about/guidelines.xhtml

(PRISMA-P) 2015 statement. *Systematic Reviews*, 4(1): 1.doi: 10.1186/2046-4053-4-1

36. Mackintosh, K. A., Chappel, S. E., Salmon, J., Timperio, A., Ball, K., Brown, H., Macfarlane S, Ridgers ND. (2019). Parental perspectives of a wearable activity tracker for children younger than 13 years: Acceptability and usability study. *JMIR mHealth uHealth*, 7(11), e13858. doi: 10.2196/13858

37. Dias, P., Brito, R., Ribbens, W., Daniela, L. (2016). The role of parents in the engagement of young children with digital technologies: Exploring tensions between rights of access and protection, from “Gatekeepers” to “Scaffolders”. *Global Studies of childhood*, 6, 414-427. doi: 10.1177/2043610616676024

38. Sekhon, M., Cartwright, M. & Francis, J. J. (2017). Acceptability of healthcare interventions: An overview of reviews and development of a theoretical framework. *BMC Health Services Research,* 17, 88. doi:10.1186/s12913-017-2031-8

39. Marston, L. (2010). *Introductory Statistics for Health and Nursing Using SPSS*. Sage Publications, Ltd.

40. Landis, J. R., & Koch, G. G. (1977). The measurement of observer agreement for categorical data. *Biometrics*, *33*(1), 159–174.

41. Critical Appraisal Skills Programme UK. (n.d.). *CASP checklists.* Retrieved from https://casp-uk.net/casp-tools-checklists/

42. Ryan, R., & Cochrane Consumers and Communication Review Group. (2013). ‘*Cochrane Consumers and Communication Review Group: data synthesis and analysis*’. Retrieved from: http://cccrg.cochrane.org.

43. Levitt H. M. (2018). How to conduct a qualitative meta-analysis: Tailoring methods to enhance methodological integrity. *Psychotherapy research : journal of the Society for Psychotherapy Research*, *28*(3), 367–378. doi:10.1080/10503307.2018.1447708

44. Thomas, J., & Harden, A. (2008). Methods for the thematic synthesis of qualitative research in systematic reviews. *BMC Medical Research Methodologies*, 1, 8-45. doi: 10.1186/1471-2288-8-45.

For peer review only - http://bmjopen.bmj.com/site/about/guidelines.xhtml

EMBASE Search terms

1. child*.ab,ti
2. exp child/
3. primary school.ab,ti.
4. youth*.ab,ti.
5. exp juvenile/
6. kindergar*en.ab,ti.
7. (school adj2 (children or kid* or pupil*)).ab,ti.
8. young people*.ab,ti
9. 1or2or3or4or5or6or7or8
10. (actigraph* or actimet* or actograp* or actomet* or acceleromet*).ab,ti.
11. "motor activity".ab,ti.
12. Fitbit.ab,ti.
13. ((electronic or remote or wearable or fitness or activity) adj2 (track* or monitor* or wearable* or device* or technolo*)).ab,ti.
14. step count.ab,ti
15. 10 or 11 or 12 or 13 or 14
16. acceptability.ab,ti.
17. experience*.ab,ti.
18. perception*.ab,ti.
19. feasibility.ab,ti.
20. feedback.ab,ti.
21. design*.ab,ti.
22. usability.ab,ti.
23. practicability.ab,ti.
24. willingness.ab,ti.
25. usefulness.ab,ti.
26. engagement.ab,ti.
27. opinion.ab.ti
28. 16 or 17 or 18 or 19 or 20 or 21 or 22 or 23 or 24 or 25 or 26 or 27
29. 9 and 15 and 28

For peer review only

Full data extraction form

Data Extraction Form		Review date:
		Ref ID:
Author, year, date		
Title		
Journal/source		
Publication type, status		
Location of study		
Qualitative/quantitative		
Aims		
Design		
Number of participants		Drop out:
Information on age		
Information on sex		
Diagnosis		
Inclusion/exclusion criteria		
Device name, description (inc. mode of delivery)		
Device purpose		
Device wear location		
Protocol wear time and intensity		Actual:
Information on protocol adherence		
Information on device engagement/incentivisation		
Acceptability assessment measures		
Description of Outcomes		
Notes		

For peer review only

PRISMA-P (Preferred Reporting Items for Systematic review and Meta-Analysis Protocols) 2015 checklist: recommended items to address in a systematic review protocol*

Section and topic	Item No	Checklist item
**ADMINISTRATIVE INFORMATION**		
Title:		
Identification	1a	Identify the report as a protocol of a systematic review
Update	1b	If the protocol is for an update of a previous systematic review, identify as such
Registration	2	If registered, provide the name of the registry (such as PROSPERO) and registration number
Authors:		
Contact	3a	Provide name, institutional affiliation, e-mail address of all protocol authors; provide physical mailing address of corresponding author
Contributions	3b	Describe contributions of protocol authors and identify the guarantor of the review
Amendments	4	If the protocol represents an amendment of a previously completed or published protocol, identify as such and list changes; otherwise, state plan for documenting important protocol amendments
Support:		
Sources	5a	Indicate sources of financial or other support for the review
Sponsor	5b	Provide name for the review funder and/or sponsor
Role of sponsor or funder	5c	Describe roles of funder(s), sponsor(s), and/or institution(s), if any, in developing the protocol
**INTRODUCTION**		
Rationale	6	Describe the rationale for the review in the context of what is already known
Objectives	7	Provide an explicit statement of the question(s) the review will address with reference to participants, interventions, comparators, and outcomes (PICO)
**METHODS**		
Eligibility criteria	8	Specify the study characteristics (such as PICO, study design, setting, time frame) and report characteristics (such as years considered, language, publication status) to be used as criteria for eligibility for the review
Information sources	9	Describe all intended information sources (such as electronic databases, contact with study authors, trial registers or other grey literature sources) with planned dates of coverage
Search strategy	10	Present draft of search strategy to be used for at least one electronic database, including planned limits, such that it could be repeated
Study records:		
Data management	11a	Describe the mechanism(s) that will be used to manage records and data throughout the review

For peer review only

Selection process	11b	State the process that will be used for selecting studies (such as two independent reviewers) through each phase of the review (that is, screening, eligibility and inclusion in meta-analysis)
Data collection process	11c	Describe planned method of extracting data from reports (such as piloting forms, done independently, in duplicate), any processes for obtaining and confirming data from investigators
Data items	12	List and define all variables for which data will be sought (such as PICO items, funding sources), any pre-planned data assumptions and simplifications
Outcomes and prioritization	13	List and define all outcomes for which data will be sought, including prioritization of main and additional outcomes, with rationale
Risk of bias in individual studies	14	Describe anticipated methods for assessing risk of bias of individual studies, including whether this will be done at the outcome or study level, or both; state how this information will be used in data synthesis
Data synthesis	15a	Describe criteria under which study data will be quantitatively synthesised
	15b	If data are appropriate for quantitative synthesis, describe planned summary measures, methods of handling data and methods of combining data from studies, including any planned exploration of consistency (such as I^2, Kendall's τ)
	15c	Describe any proposed additional analyses (such as sensitivity or subgroup analyses, meta-regression)
	15d	If quantitative synthesis is not appropriate, describe the type of summary planned
Meta-bias(es)	16	Specify any planned assessment of meta-bias(es) (such as publication bias across studies, selective reporting within studies)
Confidence in cumulative evidence	17	Describe how the strength of the body of evidence will be assessed (such as GRADE)

*** It is strongly recommended that this checklist be read in conjunction with the PRISMA-P Explanation and Elaboration (cite when available) for important clarification on the items. Amendments to a review protocol should be tracked and dated. The copyright for PRISMA-P (including checklist) is held by the PRISMA-P Group and is distributed under a Creative Commons Attribution Licence 4.0.**

From: Shamseer L, Moher D, Clarke M, Ghersi D, Liberati A, Petticrew M, Shekelle P, Stewart L, PRISMA-P Group. Preferred reporting items for systematic review and meta-analysis protocols (PRISMA-P) 2015: elaboration and explanation. BMJ. 2015 Jan 2;349(jan02 1):g7647.

For peer review only

Examining the acceptability of actigraphic devices in children using qualitative and quantitative approaches: protocol for a systematic review and meta-analysis

Journal:	*BMJ Open*
Manuscript ID	bmjopen-2022-070597.R1
Article Type:	Protocol
Date Submitted by the Author:	23-Jan-2023

Complete List of Authors:	Morris, Anna; King's College London Institute of Psychiatry Psychology and Neuroscience, Child and Adolescent Psychiatry<br>Telesia, Laurence; King's College London Institute of Psychiatry Psychology and Neuroscience, ; South London and Maudsley Mental Health NHS Trust,<br>Wickersham, Alice; King's College London, Institute of Psychiatry, Psychology and Neuroscience;<br>Epstein, Sophie; South London and Maudsley NHS Foundation Trust, NIHR Maudsley Biomedical Research Centre<br>Matcham, Faith; University of Sussex, School of Psychology<br>Sonuga-Barke, Edmund; King's College London,<br>Downs, Johnny; Kings College London, Institute of Psychiatry, Psychology and Neuroscience

**Primary Subject Heading**:	Mental health
Secondary Subject Heading:	Paediatrics, Qualitative research, Research methods
Keywords:	Child & adolescent psychiatry < PSYCHIATRY, Health informatics < BIOTECHNOLOGY & BIOINFORMATICS, Community child health < PAEDIATRICS, Systematic Review

SCHOLARONE™ Manuscripts

For peer review only - http://bmjopen.bmj.com/site/about/guidelines.xhtml

Examining the acceptability of actigraphic devices in children using qualitative and quantitative approaches: protocol for a systematic review and meta-analysis

Morris, A. C[1]., Telesia, L[1,2]., Wickersham, A [3,4]., Epstein, S[3]., Matcham, F. A. E[5]., Sonuga-Barke, E. J. S[1]., & Downs, J. M. (2021)[1,2].

[1] King's College London, London, UK, Department of Child and Adolescent Psychiatry, Institute of Psychiatry Psychology and Neuroscience; [2] South London and Maudsley NHS Foundation Trust, London, UK; [3] King's College London, London, UK, Department of Psychological Medicine, Institute of Psychiatry Psychology and Neuroscience & NIHR South London and Maudsley Biomedical Research Centre;[4] NIHR Maudsley Biomedical Research Centre, South London and Maudsley NHS Foundation Trust, London, UK, [5] School of Psychology, University of Sussex, Falmer, UK

- anna.morris@kcl.ac.uk
- laurence.telesia@kcl.ac.uk
- alice.wickersham@kcl.ac.uk
- sophie.epstein@kcl.ac.uk
- f.matcham@sussex.ac.uk
- edmund.sonuga-barke@kcl.ac.uk
- johnny.downs@kcl.ac.uk

Correspondence: Anna Morris, Department of Child and Adolescent Psychiatry, Institute of Psychiatry, Psychology and Neuroscience, King's College London, PO Box 84, De Crespigny Park, London SE5 8AF, UK; Email: anna.morris@kcl.ac.uk

Abstract

Introduction: Actigraphy is commonly used to record free-living physical activity in both typically and atypically developing children. While the accuracy and reliability of actigraphy have been explored extensively, research regarding young people's opinion towards these devices is scarce. This review aims to identify and synthesise evidence relating to the acceptability of actigraphic devices in 5-11-year olds.

Methods and analysis: Database searches will be applied to Embase, MEDLINE, PsychInfo and Social Policy and Practice through the OVID interface; and Education

For peer review only - http://bmjopen.bmj.com/site/about/guidelines.xhtml

For peer review only

Resources Information Center (ERIC), British Education Index and CINAHL through the EBSCO interface from January 2018 until the present. Supplementary forward and backward citation and grey literature database searches, including Healthcare Management Information Consortium (HMIC) and PsycEXTRA will be conducted. Qualitative and quantitative studies, excluding review articles and meta-analyses, will be eligible, without date restrictions. Article screening and data extraction will be undertaken by two review authors and disagreements will be deferred to a third reviewer. The primary outcome, actigraphic acceptability, will derive from the narrative synthesis of the main themes identified from included qualitative literature, and pooled descriptive statistics relating to acceptability identified from quantitative literature. Subgroup analyses will determine if acceptability changes as a function of the key participant and actigraphic device factors.

Ethics and dissemination: Ethical approval is not required for this systematic review as it uses data from previously published literature. The results will be presented in a manuscript and published in a peer-review journal and will be considered alongside a separate stream of co-design research to inform the development of a novel child-worn actigraphic device.

PROSPERO Registration number: CRD42021232466

Article Summary

- Detailed study methodology and statistical analysis strategy are planned, to enable the inclusion of quantitative and qualitative literature.
- Comprehensive search terms are informed by a theoretical framework to enhance the likelihood of detecting literature relevant to acceptability.
- Literature not written in English will be excluded and may lead pertinent studies to being omitted.
- Conclusions that can be surmised from the evidence of this systematic review could be restricted by limited actigraph monitoring protocol adherence and attrition data reported in quantitative studies and the potentially low quality of identified literature.
- Narrative synthesis of qualitative evidence may be influenced by reviewer subjectivity and must be critically appraised.

Background

For peer review only - http://bmjopen.bmj.com/site/about/guidelines.xhtml

Actigraphy, an objective measure of movement, is widely considered the preferred method for activity-based monitoring in clinical and epidemiological research. [1] Modern actigraphic devices, known as actigraphs are small, inexpensive, and readily accessible rendering them ideal for inclusion in studies investigating free-living patterns of physical activity and sleep-wake cycles. [2] Actigraphs are credited for their ability to overcome important measurement biases associated with traditional subjective methods including memory, perception, and social desirability bias to produce a more detailed and accurate account of motor activity. [3] These benefits are particularly pronounced in the study of young children, where parent or teacher-reported questionnaires are often relied upon as the primary source of information, [4] and where self-reported measures tend to be inaccurate. [5, 6]

Actigraphs have been applied extensively in the remit of children's physical and mental health, especially in the context of sleep medicine, where actigraphic devices are used to record nocturnal movement patterns in children. [7] Actigraphic devices are increasingly relied upon to examine sleep patterns in childhood neurological disorders [8] and neurodevelopmental disorders. [9, 10] Deficiencies in sleep quantity or quality are also linked to the onset of various health difficulties, [11, 12] behavioural problems, and cognitive impairments. [13] Therefore, objective measures of sleep are increasingly used to support clinical assessment and management of sleep disturbances or disorders. [4] Compared to more costly data collection methods such as polysomnography which typically records sleep information for one or two nights in a laboratory, actigraphy can be used in naturalistic environments for extended periods, [14] making it easier to collect data from children who might find it difficult to sleep normally in unfamiliar surroundings. [15, 16]

Considerable efforts have also been made to deploy actigraphs to assess physical activity in children, including obesity, [17, 18] and chronic conditions such as cerebral palsy. [19] Particular attention is also being paid to the potential role of actigraphy in the assessment and diagnosis of Attention Deficit Hyperactivity Disorder (ADHD), [20] a common childhood disorder characterised by the persistent presence of three core symptoms, namely impulsivity, inattention, and hyperactivity. [21, 22] Within clinical practice, diagnosis of ADHD is usually based upon a clinical assessment of the individual, supplemented by collateral history and reports from parents/carers and teachers. [21, 23, 24, 25] However, the clinical utility of these measures has been questioned, as the main features of the disorder are rarely observed directly or systematically measured by members of the clinical decision-making team. [26] In contrast, actigraphy provides the means to objectively monitor physical activity, a well-established means of determining the severity of hyperactive symptoms, [27] across a variety of different contexts.

For peer review only - http://bmjopen.bmj.com/site/about/guidelines.xhtml

There is a considerable body of evidence focusing on the accuracy of actigraphic outcomes in the context of physical activity and sleep, [28, 29, 30] however, the question of acceptability has often been overlooked. Engagement with medical devices is heavily dependent on aesthetic preferences [31] and is potentially even more pertinent for children, who may have unique physical feature requirements compared to adults. [32] Children with neurodevelopmental disorders may also experience particular tactile sensitivities associated with the actigraphic device or its placement, [33] and stigma associated with visible markers of medical investigation. [34] Therefore, it is essential to design actigraphs that incorporate the needs and preferences of children to ensure sustained engagement with these products, particularly when asking children to wear them continuously over prolonged periods in-home and school-based environments.

Aims

This review aims to examine the literature pertaining to the acceptability of body-worn actigraphic devices for children aged 5-11 years old. Whilst this review may discover overlap between features considered acceptable by both typically and atypically developing children, differences between these groups will be highlighted if identified. If available, we aim also to include feedback on acceptability from parents/caregivers of young children who may provide insightful feedback about the acceptability of these devices. As such, the results of this review may have several practical applications, both in typically and atypically developing children. For example, by offering information to clinical teams on the types of actigraphic devices that are likely to be well tolerated by children under their care or guiding the development of future study protocols to include child-accepted actigraphs.

Further, we aim to use the findings from this review alongside data gathered from concurrent co-design research, to inform the design of a bespoke actigraphic device primarily indented to monitor physical activity within this age group.

Methods and analysis

This protocol has been prospectively recorded in the PROSPERO international database. We used the Preferred Reporting Items for Systematic Review and Meta-Analysis protocols (PRISMA-P; online supplementary file 1) checklist when writing our report. [35]

For peer review only - http://bmjopen.bmj.com/site/about/guidelines.xhtml

Eligibility criteria

Population

We are principally interested in actigraphic acceptability among primary school-aged children. In the UK, this represents children aged 5-11, an age range that typically encompasses most primary school-aged children worldwide. Therefore, studies will be included in this review if they involve children (where at least 50% fall within the 5-11 year age range or where the average age falls within this age range) in the design, development, or evaluation of an actigraphic device. Additionally, parents and caregivers of young children often act as the 'gatekeeper' responsible for enabling or restricting access to or time spent interacting with wearable technologies, [36, 37] therefore, studies including parents or caregiver/s of children aged between 5-11 years old will also be included.

Exposure and outcome

Studies will be eligible for this review if they investigate the development or use of an actigraph and report a corresponding measure of acceptability. In this review, acceptability will be defined according to the Theoretical Framework of Acceptability (TFA), [38] including participants' affective (e.g. feelings), cognitive (e.g. perceptions) or behavioural (e.g. attrition rates) response towards the actigraph. Included studies may use actigraphs for any purpose, as the primary focus of the study or as part of a multifaceted tool. Participants must be required to wear an actigraphic device in a community setting to be included in this review. Studies will be excluded if they required the wearable device to be worn in only a lab-based setting.

Study design

Qualitative studies, specifically co-design, focus groups and interview methodologies will be included as well as quantitative studies including feasibility, pilot, observational cohort, or intervention studies. Reviews, meta-analyses, and protocols will be excluded, as will commentaries, opinion pieces, editorials, magazine articles, and technical or internal reports. As patient-centred design and acceptability literature is an emerging field we will extend inclusion to published conference abstracts if they provide sufficient information to demonstrate that they meet the inclusion criteria. Likewise, letters that describe the methods and results of otherwise unpublished research may also be considered for

For peer review only - http://bmjopen.bmj.com/site/about/guidelines.xhtml

inclusion. Searches will be conducted from January 2018 until the present as this timeframe will capture the recent rapid expansion of wearable technology but is also likely to include publications which use older devices that are still suitable for use and potentially pertinent to current-day children. Studies can be published or unpublished but must be written in English. See table 1 for more detailed inclusion and exclusion criteria.

Table 1. Inclusion/exclusion table for studies exploring actigraphic devices in children.

	Inclusion criteria	Exclusion criteria
Publication status	Studies written in English<br>Published or unpublished studies	Studies written in other languages<br>Commentaries, opinion pieces, editorials, magazine articles and technical or internal reports
Study design	Qualitative or quantitative studies	Reviews or meta-analyses
Device	Studies in which a wearable activity tracker to measure motor activity was worn by a child within the target age group	Studies which do not employ any wearable devices<br>Studies using wearable devices which measure parameters other than motor activity (e.g., balance, number of steps, proportion of time sitting etc.)<br>Studies in which wearable activity trackers were discussed, but not worn
Age	Studies where all participants are aged between 5-11 years, as indicated by age range, or reported school year	Studies where all participants are outside the target age of 5-11 years

For peer review only - http://bmjopen.bmj.com/site/about/guidelines.xhtml

	Studies reporting a measure of central tendency (e.g., median, mean) which falls within the range of 5-11 years<br>Studies that present data in a way that allows extraction for a subgroup aged between 5-11 years<br>Studies that satisfy the above age criteria for the child wearing the device, but feedback is provided by a caregiver or other relevant stakeholder	Studies where it is not possible to extract data specific for participants aged between 5-11 years
Setting	Studies with protocols which involves the wear of an activity tracker in a community setting, regardless of whether the device is also worn in a non-community setting	Studies which take place entirely in a non-community setting (e.g., laboratory, university or hospital setting)
Acceptability	Studies which present a qualitative or quantitative measure of acceptability<br>Measures can include focus group or interview feedback, proportions of participants declining to wear the device or proportion and extent of deviation from the study's wear protocol	Studies which do not contain any direct or indirect measure of acceptability

Information sources and search strategy

The electronic databases Embase, MEDLINE, PsychInfo and Social Policy and Practice through the OVID interface; and Education Resources Information Center (ERIC), British

For peer review only - http://bmjopen.bmj.com/site/about/guidelines.xhtml

Education Index and CINAHL through the EBSCO interface will be searched using a combination of free text and thesaurus terms.

The search terms will derive from three key concepts, children (population), actigraphy (exposure) and acceptability (outcome). Search terms and advanced search techniques will be utilised, including truncation, wild card and adjacency operators, which will be tailored for each database as required. We will also review the reference list of included articles and related existing review articles (backward citation searching), and articles which have referenced included studies (forward citation searching). Both forward and backward citation searching will be conducted in Web of Science Core Collection, and if a citation is unavailable in this source, Google Scholar will be queried.

Additional grey literature searches will be conducted within PsycEXTRA, a complementary database to PsychINFO and the Healthcare Management Information Consortium (HMIC), comprising records from the Department of Health (DoH) and the King's Fund. Supplementary searches will be carried out using the same search strategy described above for the primary electronic databases. In addition, consultation with experts will be conducted and authors of studies included in the review will be contacted to inquire about any known studies matching the inclusion criteria. Full search strategies for the database are provided as follows; Embase (online supplementary file 2), MEDLINE (online supplementary file 3), PsychInfo (online supplementary file 4), Social Policy and Practice (online supplementary file 5), ERIC (online supplementary file 6), British Education Index (online supplementary file 7) and CINAHL (online supplementary file 8).

Data management

The search output will be downloaded into EndNote version 20 reference management software. Duplicates will automatically be detected and then removed by the reviewers. The remaining references will be exported to a Microsoft Excel document and manually screened for undetected duplicates.

Study selection process

Two reviewers (AM and LT) will each independently screen a 10% sample of the titles and abstracts generated by the search strategy. Interrater reliability for this sample will be determined using Cohen's kappa. [39] If the interrater reliability is considered "substantial" or "almost perfect", based on a kappa statistic greater than 0.60 [40] each review author will

For peer review only - http://bmjopen.bmj.com/site/about/guidelines.xhtml

independently review 50% of the remaining abstracts, with the option to jointly discuss unclear or otherwise challenging abstracts. If the strength of agreement for the 10% overlap sample is lower, both authors will review all remaining abstracts. Any disagreements between the reviewers regarding suitability for inclusion will be rectified by joint decision and, if the dispute continues, a third reviewer (JD) will decide. Full-text screening will be performed for studies deemed eligible or of unclear eligibility, again by the same two independent reviewers and a third reviewer where disagreement regarding inclusion persists. AM and LT will perform backward and forward citation searches to identify any other relevant literature. To establish agreement, this process will be conducted by both review authors (AM & LT) for 10% of the included reference lists, and the remainder evenly distributed between the authors for independent screening. Further study inclusion will be determined using the previously described consensus approach. All article exclusions will be recorded and will be justified for any articles removed at the full-text stage.

Data items and extraction process

Each paper selected for inclusion will be reviewed and data contained within the paper will be extracted and recorded using a data extraction form adapted from the Cochrane Review Group Data Extraction Form (online supplementary file 9). Both review authors (AM & LT) will perform data extraction and any disagreements regarding this procedure will be discussed and settled by a third reviewer (JD) if required. Before starting, extraction consistency will be examined by piloting the form with 10% of eligible studies. Any disagreements arising from this process will be resolved through discussion between review authors (AM and LT), and a third reviewer will decide on unresolved disagreements (JD). If needed, supplementary information will be requested directly from study authors to finalise inconsistencies and complete extraction.

For both qualitative and quantitative papers, extracted data will include - study characteristics (e.g. aims, design, sample size and analysis), information source (e.g. journal article or conference abstract), country and date of origin, title, and publication status. Participant descriptive data, including age, gender, and mental/physical health status will be recorded. Where provided, details regarding the actigraphic device will also be recorded, including device name, purpose (e.g. sleep, obesity, ADHD symptom tracking), features (e.g. material, design and functionality), body-worn location and mode of delivery (e.g. on its own or part of a multi-faceted tool).

All text relating to relevant qualitative findings will be collected from the results section of included papers, to record themes and sub-themes pertaining to child-worn actigraph

For peer review only - http://bmjopen.bmj.com/site/about/guidelines.xhtml

acceptability and engagement. Particular attention will be paid to any design or contextual factors that hinder or promote actigraphic device use in the target population. Where available, this will include direct participant quotes to afford greater data granularity at the data synthesis stage. Additionally, descriptions of the research methods used to assess acceptability (e.g. structured questionnaire with space to report free text, structured or semi-structured interview or focus groups) and the information reporting source (e.g. child, caregiver, or teacher) will be collected.

Where provided, actigraph monitoring protocol adherence data will be extracted - that is the proportion of time in the given measurement period that the actigraphic device was actually worn for the duration specified in the study methods, according to self-report e.g. diary entry, or activity monitoring device data. We will also extract absolute numbers for the duration and intensity of prescribed and actual wear time, to investigate adherence according to protocol differences. Attrition rates and protocol deviations, that is the percentage of consented participants who stop wearing the study device or wore the device in a different way than directed within the observed timeframe, as well as any comments for this behaviour will be captured. Where studies also use questionnaires, scales, or other methods to ascertain acceptability/useability, the name and content of the method, as well as participants' responses will be recorded. Information on factors which could influence participant device engagement will also be extracted. This could include but is not limited to, frequency of contact with the research team, explicit reminders to wear the device, for example, phone calls from the research team or automated text or email reminders and incentivisation. As this review focuses on the acceptability rather than the effectiveness of actigraphs, data relating to changes in behavioural outcomes following the use of an actigraphic device (e.g. improved sleep or increased physical activity) will not be collected.

If a study is found which includes at least 50% of the children aged 5-11 years as well as a minority outside of this age group, we will aim to extract data related only to the age group of interest. If it is not possible to do so, this will be highlighted in the finalised report.

Quality assessment

The relevant Critical Appraisal Skills Programme (CASP) UK [41] checklists will be used to assess the quality of included articles according to study design. Quality assessment for each paper included in the review will be independently conducted by two review authors (AM and LT), and any appraisal discrepancies will be resolved by a third reviewer (JD). Studies will not be excluded based on methodological quality, as the primary aim of this review is to identify literature about end users' experiences interacting with actigraphs.

For peer review only - http://bmjopen.bmj.com/site/about/guidelines.xhtml

Rather, the appraisal will be used to comment on the general quality of available literature regarding the acceptability of body-worn actigraphic devices in children.

Data synthesis and analysis

This review will utilise a systematic narrative synthesis approach. [42] The characteristics and key results for all included studies will first be presented in a table and elaborated on in the main body of the text. Summarised data will include, study aims, the types of actigraphic devices, details about the participant group and specific constructs of acceptability that were assessed.

For qualitative studies, the main themes, data collection method and analysis method (e.g. thematic, grounded theory or content analysis) will be presented. As outlined by Levitt [43], to meta-analyse the qualitative studies, data relevant to the review question will be selected from the included studies to form "meaning units" which will be compared with those from other studies to develop common themes and higher-order categories. Depending on the number and type of papers identified by the search, it may be more appropriate to simply summarise the findings at this stage. However, to allow meaningful comparisons, where possible the results will also be presented by subgroups, that is, according to device types or specific participant factors to better surmise any aesthetic requirements that are shared between or are unique to certain populations. Potential subgroups include, but will not be limited to, whether the children of interest are typically or atypically developing or have been diagnosed with a mental or physical health condition. Similarly, as the eligibility criteria span a period of considerable developmental change, the patterns of acceptability by age will be explored, as well as gender and ethnicity.

Descriptive statistics relating to acceptability will be summarised for quantitative studies, including percentages, frequencies, and measures of central tendency. If multiple studies are included that overlap sufficiently in methodology and outcome variables, random effects meta-analytical procedures will be used. For example, if possible, a pooled estimate of proportional wear time will be produced accompanied by a 95% confidence interval (CI) using the Stata command Metaprop. The same procedure will be applied where two or more studies employ the same acceptability questionnaires or other outcome measures. For example, if compliance data is presented as a binary outcome, such as a frequency count of participants deviating from prescribed wear protocol or proportion failing to achieve a minimum wear-time, the relative risk for that outcome will be calculated and meta-analytic methods for dichotomous outcomes will be considered, Statistical heterogeneity will be reported using the I^2 statistic. If ten or more studies are identified for inclusion within a meta-

For peer review only - http://bmjopen.bmj.com/site/about/guidelines.xhtml

analysis, techniques will be used to assess publication bias where appropriate, including Egger's regression test and funnel plots. Where possible, results stratified by actigraphic device type will be determined, and if the data permits, subgroup analyses will be reported according to the participant variables described for the qualitative subgroup analyses strategy.

Patient and public involvement

No patients were involved in the conception or development of this project.

Discussion

This systematic review seeks to identify and summarise the existing literature relating to actigraphic device acceptability in young children and highlight particular features that make actigraphic products more or less palatable in childhood. To our knowledge, this is the first synthesis to focus on child and parental acceptance of actigraph use in free-living settings for this age group, despite the potential impact of acceptability on wear time. [30, 32]

Our intention to include studies which focus on both the subjective, experiential reports from children and caregivers, as well as objective markers of wear time and attrition will allow a more comprehensive understanding of the role particular features within actigraphs play. For example, if qualitative reports consistently raise a feature as relevant, it may be possible to use a quantitative measure to corroborate this by comparing studies using devices with that feature to those without. Conversely, this approach may contradict the qualitative reports, thus identifying areas for further exploration and future research.

We hope that, by including studies with a range of prescribed wear times as well as focusing primarily on community settings, our findings will have an impact on real-world research and clinical applications. For example, some features may promote compliance in the short term only, whereas another feature is associated with better prolonged wear. This knowledge, along with an understanding of the preferences of specific subgroups, would allow clinicians and researchers to select the optimal device for their needs, and support designers to develop new products targeted to specific applications. This is timely given the rise of commercially available actigraphs purposefully developed for this age group. [36]

Furthermore, by collating and meta-analysing wear time and attrition data from several studies we hope to set a benchmark, against which new devices may be compared. Such a

For peer review only - http://bmjopen.bmj.com/site/about/guidelines.xhtml

benchmark could be helpful to device designers, particularly those wishing to evaluate prototypes against existing models in an iterative design process.

To strengthen this review, we have used a theoretical framework, the TFA to develop the search strategy, as well as use broad search terms to maximise the chances of identifying papers relevant to the study question. A possible limitation is that the process of deducing themes across varied qualitative studies has been criticised owing to the potential interference of reviewer subjectivity on the study findings. [44] However, we have brought together a team of reviewers from multi-disciplinary backgrounds to minimise any undue influence from any one reviewer's judgment. Our decision to exclude non-English written literature may limit the number of papers identified but is necessary due to resource constraints.

Ethics and dissemination

This systematic review will use existing data and therefore does not require ethical approval. Findings from the review will be published in a peer-review journal and used to inform the development of a new actigraphic device.

Footnotes:

Contributors: AM (corresponding author) and LT coconceived and developed the review protocol, defined the search strategy, drafted the initial, revised, and finalised manuscripts. AM and LT will perform data extraction and analysis and interpret the review findings. AW and SE provided critical revisions to the study design and manuscript. JD, ESB and FM coconceived the review, supported the study design and amendments to the manuscript.

Funding statement:

This review is funded by the National Institute for Health Research (NIHR) Clinician Science Fellowship award (CS-2018-18-ST2-014). LT is funded by the NHIR as an Academic Clinical Fellow. AW is in receipt of a PhD studentship funded by the NIHR Biomedical Research Centre (BRC) at South London and Maudsley NHS Foundation Trust and Kings College London (NIHR-INF-0690). SE is funded by a Medical Research Council (MRC) Clinical Research Training Fellowship (MR/T001437/1) and previously received salary support from an MQ Data Science Award and from the Psychiatry Research Trust. JD is funded by the Clinician Science Fellowship award (CS-2018-18-ST2-014) and received additional funding from an MRC Clinical Research Training Fellowship (MR/L017105/1) and Psychiatry

For peer review only - http://bmjopen.bmj.com/site/about/guidelines.xhtml

Research Trust Peggy Pollak Research Fellowship in Developmental Psychiatry. ESB is funded by the BRC, MRC (MR/T046864/1), Economic and Social Research Council (ESRC; ES/S004467/1), UK Research and Innovation's (UKRI) ESRC (ES/V016393/1) and NIHR Programme Grants for Applied Research (PGfAR; RP-PG-0618-20003).

Disclaimer: This paper represents independent research [part] funded by the NIHR Biomedical Research Centre at South London and Maudsley NHS Foundation Trust and King's College London. The funding body has not been involved in the design of the study, the collection or analysis of data, or the interpretation of data.

Competing interests statement: None declared.

References:

1. Migueles, J. H., Cadenas-Sanchez, C., Ekelund, U., Delisle Nyström, C., Mora-Gonzalez, J., Löf, M., Labayen, I., Ruiz, J. R., & Ortega, F. B. (2017). Accelerometer data collection and processing criteria to assess physical activity and other outcomes: A systematic review and practical considerations. *Sports Medicine*, 47(9),1821-1845. doi: 10.1007/s40279-017-0716-0.

2. Martin, J. L., & Hakim, A. D. (2011). Wrist actigraphy. *Chest*, 139(6), 1514-1527. doi: 10.1378/chest.10-1872.

3. Arvidsson, D., Fridolfsson, J., & Borjesson, M. (2019). Measurement of physical activity in clinical practice using accelerometers. *Journal of Internal Medicine*, 286, 137–153. doi:10.1111/joim. 12908

4. Mazza, S., Bastuji, H., & Rey, A. E. (2020). Objective and subjective assessments of sleep in children: Comparison of actigraphy, sleep diary completed by children and parents' estimation. *Frontiers in psychiatry*, 11, 495. doi: 10.3389/fpsyt.2020.00495

5. Adamo, K. B., Prince, S. A., Tricco, A. C., Connor-Gorber, S., & Tremblay, M. A. (2009). Comparison of indirect versus direct measures for assessing physical activity in the pediatric population: A systematic review, *International Journal of Pediatric Obesity*, 4:1, 2-27. doi: 10.1080/17477160802315010

6. Chinapaw, M. J. M., Mokkink, L. B., van Poppel, M. N. M., van Mechelen, W., & Terwee, C. B. (2010). Physical activity questionnaires for youth: A systematic review

For peer review only - http://bmjopen.bmj.com/site/about/guidelines.xhtml

of measurement properties. *Sports Medicine*, 40 (7), 539-563. doi:10.2165/11530770-000000000-00000.

7. Meltzer, L. J., Montgomery-Downs, H. E., Insana, S. P., & Walsh, C. M. (2012). Use of actigraphy for assessment in pediatric sleep research. *Sleep Medicine Reviews*, 16(5):463-75. doi: 10.1016/j.smrv.2011.10.002.

8. Tsai, S. Y., Lee, W. T., Jen, S. F., Lee, C., & Weng, W. C. (2018). Sleep and behavior problems in children with epilepsy. *Journal of Pediatric Health Care*, 18, 30123-8. doi: 10.1016/j.pedhc.2018.07.004

9. Merbler, A. M., Byiers, B. J., Garcia, J. J., Feyma, T. J., & Symons, F. J. (2018). The feasibility of using actigraphy to characterize sleep in Rett syndrome. *Journal of Neurodevelopmental Disorders*, 10(1), 8. doi: 10.1186/s11689-018-9227-z.

10. De Crescenzo, F., Licchelli, S., Ciabattini, M., Menghini, D., Armando, M., Alfieri, P., Mazzone, L., Pontrelli, G., Livadiotti, S., Foti, F., Quested, D., & Vicari, S. (2016). The use of actigraphy in the monitoring of sleep and activity in ADHD: A meta-analysis. *Sleep medicine reviews*, *26*, 9–20. doi: 10.1007/s12402-013-0122-x

11. Cappuccio, F. P., Taggart, F. M., Kandala, N. B., Currie, A., Peile, E., Stranges, S., & Miller, M. A. (2008). Meta-analysis of short sleep duration and obesity in children and adults. *Sleep*, 31, 619–26. doi:10.1093/sleep/31.5.619

12. Smaldone, A., Honig, J. C., & Byrne, M. W. (2007). Sleepless in America: Inadequate sleep and relationships to health and well-being of our nation's children. *Pediatrics*, 119, S29–37. doi:10.1542/peds.2006-2089F.

13. Maski, K. P., & Kothare, S. V. (2013). Sleep deprivation and neurobehavioral functioning in children. *International Journal of Psychophysiology*, 89, 259–64. doi: 10.1016/j.ijpsycho.2013.06.019.

14. Verhulst, S. L., Schrauwen, N., De Backer, W. A., & Desager, K. N. (2006). First night effect for polysomnographic data in children and adolescents with suspected sleep disordered breathing. *Archives of Disease in Childhood*, 91, 233–7. doi:10.1136/adc.2005.085365.

For peer review only - http://bmjopen.bmj.com/site/about/guidelines.xhtml

15. Sadeh, A., Alster, J., Urbach, D., & Lavie, P. (1989). Actigraphically based automatic bedtime sleep-wake scoring: Validity and clinical applications. *Journal of Ambulatory Monitoring*, 2, 209–216.

16. Sadeh, A., Lavie, P., Scher, A., Tirosh, E., & Epstein, R. (1991). Actigraphic home-monitoring sleep-disturbed and control infants and young children: A new method for pediatric assessment of sleep-wake patterns. *Pediatrics*, 87, 494–499.

17. Jiménez-Pavón, D., Kelly, J., & Reilly, J. J. (2010). Associations between objectively measured habitual physical activity and adiposity in children and adolescents: Systematic review. *International Journal of Pediatric Obesity*, 5(1), 3-18. doi: 10.3109/17477160903067601.

18. Wilks, D. C., Besson, H., Lindroos, A. K., Ekelund, U. (2011). Objectively measured physical activity and obesity prevention in children, adolescents and adults: A systematic review of prospective studies. *Obesity Reviews*, 12(5), e119-29. doi: 10.1111/j.1467-789X.2010.00775.x.

19. Keawutan, P., Bell, K., Davies, P. S., & Boyd, R. N. (2014). Systematic review of the relationship between habitual physical activity and motor capacity in children with cerebral palsy. *Research in Developmental Disabilities*, 35(6), 1301-9. doi: 10.1016/j.ridd.2014.03.028.

20. Subcommittee on Attention-Deficit/Hyperactivity Disorder, Steering Committee on Quality Improvement and Management, Wolraich, M., Brown, L., Brown, R. T., DuPaul, G., Earls, M., Feldman, H. M., Ganiats, T. G., Kaplanek, B., Meyer, B., Perrin, J., Pierce, K., Reiff, M., Stein, M. T., & Visser, S. (2011). ADHD: Clinical practice guideline for the diagnosis, evaluation, and treatment of attention-deficit/hyperactivity disorder in children and adolescents. *Pediatrics*, *128*(5), 1007–1022. doi:10.1542/peds.2011-2654.

21. De Crescenzo, F., Armando, M., Mazzone, L., Ciliberto, M., Sciannamea, M., Figueroa, C., Janiri, L., Quested, D., & Vicari, S. (2014). The use of actigraphy in the monitoring of methylphenidate versus placebo in ADHD: A meta-analysis. *Attention deficit and hyperactivity disorders*, *6*(1), 49–58. doi: 10.1007/s12402-013-0122-x.

For peer review only - http://bmjopen.bmj.com/site/about/guidelines.xhtml

22. National Institute for Health & Care Excellence. (2018). *Attention Deficit Hyperactivity Disorder: Diagnosis and Management of ADHD in Children, Young People and Adults (National Clinical Practice Guideline Number 87)*. Retrieved from: https://www.nice.org.uk/guidance/ng87/resources/attention-deficit-hyperactivity-disorder-diagnosis-and-management-pdf-1837699732933

23. American Psychiatric Association. (2013). *Diagnostic and statistical manual of mental disorders (5th ed.).* Washington, D.C.: American Psychiatric Association

24. McConaughy, S. H., Ivanova, M., Antshel, K., Eiraldi, R. B., & Dumenci, L. (2009). Standardized observational assessment of Attention Deficit Hyperactivity Disorder combined and predominantly inattentive subtypes: II. Classroom observations. *School Psychology Review*, 39, 362–381.

25. Wolraich, M. L., Chan, E., Froehlich, T., Lynch, R. L., Bax, A., Redwine, S. T., Ihyembe, D., & Hagan, J. F. Jr. (2019). ADHD diagnosis and treatment guidelines: A historical perspective. *Pediatrics*, 144(4), e20191682. doi: 10.1542/peds.2019-1682.

26. Hall, C. L., Taylor, J. A., Newell, K., Baldwin, L., Sayal, K., & Hollis, C. (2016). The challenges of implementing ADHD clinical guidelines and research best evidence in routine clinical care settings: Delphi survey and mixed-methods study. *BJPsych Open*, 2(1), 25-31. doi: 10.1192/bjpo.bp.115.002386

27. Hall, C. L., Valentine, A. Z., Groom, M. J., Walker, G. M., Sayal, K., Daley, D., Hollis, C. (2016). The clinical utility of the continuous performance test and objective measures of activity for diagnosing and monitoring ADHD in children: A systematic review. *European Child and Adolescent Psychiatry*, 25(7), 677-99. doi: 10.1007/s00787-015-0798-x.

28. Kam, H. J., Lee, K., Cho, S. M., Shin, Y. M., Park, R. W. (2011). High-resolution actigraphic analysis of ADHD: A wide range of movement variability observation in three school courses - a pilot study. *Healthcare Informatics Research*, 17(1), 29-37. doi: 10.4258/hir.2011.17.1.29.

29. Evenson, K. R., Goto, M. M., & Furberg, R. D. (2015). Systematic review of the validity and reliability of consumer-wearable activity trackers. *The international*

For peer review only - http://bmjopen.bmj.com/site/about/guidelines.xhtml

journal of behavioral nutrition and physical activity, 12, 159. doi: https://doi.org/10.1186/s12966-015-0314-1

30. Scott, H., Lack, L., & Lovato, N. (2020). A systematic review of the accuracy of sleep wearable devices for estimating sleep onset. *Sleep medicine reviews*, 49, 101227. doi: 10.1016/j.smrv.2019.101227.

31. O'Connor, S., Hanlon, P., O'Donnell, C. A., Garcia, S., Glanville, J., & Mair, F. S. (2016). Understanding factors affecting patient and public engagement and recruitment to digital health interventions: A systematic review of qualitative studies. *BMC Medical Information Decision Making*. 16(1), 120. doi: 10.1186/s12911-016-0359-3

32. Geljins, A. C., Killelea, B., Vitale, M., Mankad, V., & Moskowitz. A. (2006). Appendix C. The dynamics of pediatric device innovation: putting evidence in context. Safe Medical Devices for Children. Washington, DC: *The National Academies Press*. doi: 17226/11313

33. Fawkes, D. B., Malow, B. A., Weiss, S. K., Reynolds, A. M., Loh, A., Adkins, K. W., Wofford, D. D., Wyatt, A. D., Goldman, S. E. (2014). Conducting actigraphy research in children with neurodevelopmental disorders-a practical approach. *Behavioral Sleep Medicine*,12, 116. doi: 10.1080/15402002.2013.854245.

34. Mueller, A. K., Fuermaier, A. B., Koerts, J., & Tucha, L. (2012). Stigma in attention deficit hyperactivity disorder. *Attention Deficit Hyperactivity Disorder*. 4(3),101-14. doi: 10.1007/s12402-012-0085-3.

35. Moher, D., Shamseer, L., Clarke, M., Ghersi, D., Liberati, A., Petticrew, M., et al. (2015). Preferred reporting items for systematic review and meta-analysis protocols (PRISMA-P) 2015 statement. *Systematic Reviews*, 4(1): 1.doi: 10.1186/2046-4053-4-1

36. Mackintosh, K. A., Chappel, S. E., Salmon, J., Timperio, A., Ball, K., Brown, H., Macfarlane S, Ridgers ND. (2019). Parental perspectives of a wearable activity

For peer review only - http://bmjopen.bmj.com/site/about/guidelines.xhtml

tracker for children younger than 13 years: Acceptability and usability study. *JMIR mHealth uHealth*, 7(11), e13858. doi: 10.2196/13858

37. Dias, P., Brito, R., Ribbens, W., Daniela, L. (2016). The role of parents in the engagement of young children with digital technologies: Exploring tensions between rights of access and protection, from “Gatekeepers” to “Scaffolders”. *Global Studies of childhood*, 6, 414-427. doi: 10.1177/2043610616676024

38. Sekhon, M., Cartwright, M. & Francis, J. J. (2017). Acceptability of healthcare interventions: An overview of reviews and development of a theoretical framework. *BMC Health Services Research,* 17, 88. doi:10.1186/s12913-017-2031-8

39. Marston, L. (2010). *Introductory Statistics for Health and Nursing Using SPSS*. Sage Publications, Ltd.

40. Landis, J. R., & Koch, G. G. (1977). The measurement of observer agreement for categorical data. *Biometrics*, *33*(1), 159–174.

41. Critical Appraisal Skills Programme UK. (n.d.). *CASP checklists.* Retrieved from https://casp-uk.net/casp-tools-checklists/

42. Ryan, R., & Cochrane Consumers and Communication Review Group. (2013). ‘*Cochrane Consumers and Communication Review Group: data synthesis and analysis’*. Retrieved from: http://cccrg.cochrane.org.

43. Levitt H. M. (2018). How to conduct a qualitative meta-analysis: Tailoring methods to enhance methodological integrity. *Psychotherapy research : journal of the Society for Psychotherapy Research*, *28*(3), 367–378. doi:10.1080/10503307.2018.1447708

44. Thomas, J., & Harden, A. (2008). Methods for the thematic synthesis of qualitative research in systematic reviews. *BMC Medical Research Methodologies*, 1, 8-45. doi: 10.1186/1471-2288-8-45.

For peer review only - http://bmjopen.bmj.com/site/about/guidelines.xhtml

PRISMA-P (Preferred Reporting Items for Systematic review and Meta-Analysis Protocols) 2015 checklist: recommended items to address in a systematic review protocol*

Section and topic	Item No	Checklist item
**ADMINISTRATIVE INFORMATION**		
Title:		
Identification	1a	Identify the report as a protocol of a systematic review
Update	1b	If the protocol is for an update of a previous systematic review, identify as such
Registration	2	If registered, provide the name of the registry (such as PROSPERO) and registration number
Authors:		
Contact	3a	Provide name, institutional affiliation, e-mail address of all protocol authors; provide physical mailing address of corresponding author
Contributions	3b	Describe contributions of protocol authors and identify the guarantor of the review
Amendments	4	If the protocol represents an amendment of a previously completed or published protocol, identify as such and list changes; otherwise, state plan for documenting important protocol amendments
Support:		
Sources	5a	Indicate sources of financial or other support for the review
Sponsor	5b	Provide name for the review funder and/or sponsor
Role of sponsor or funder	5c	Describe roles of funder(s), sponsor(s), and/or institution(s), if any, in developing the protocol
**INTRODUCTION**		
Rationale	6	Describe the rationale for the review in the context of what is already known
Objectives	7	Provide an explicit statement of the question(s) the review will address with reference to participants, interventions, comparators, and outcomes (PICO)
**METHODS**		
Eligibility criteria	8	Specify the study characteristics (such as PICO, study design, setting, time frame) and report characteristics (such as years considered, language, publication status) to be used as criteria for eligibility for the review
Information sources	9	Describe all intended information sources (such as electronic databases, contact with study authors, trial registers or other grey literature sources) with planned dates of coverage
Search strategy	10	Present draft of search strategy to be used for at least one electronic database, including planned limits, such that it could be repeated
Study records:		
Data management	11a	Describe the mechanism(s) that will be used to manage records and data throughout the review

For peer review only

Selection process	11b	State the process that will be used for selecting studies (such as two independent reviewers) through each phase of the review (that is, screening, eligibility and inclusion in meta-analysis)
Data collection process	11c	Describe planned method of extracting data from reports (such as piloting forms, done independently, in duplicate), any processes for obtaining and confirming data from investigators
Data items	12	List and define all variables for which data will be sought (such as PICO items, funding sources), any pre-planned data assumptions and simplifications
Outcomes and prioritization	13	List and define all outcomes for which data will be sought, including prioritization of main and additional outcomes, with rationale
Risk of bias in individual studies	14	Describe anticipated methods for assessing risk of bias of individual studies, including whether this will be done at the outcome or study level, or both; state how this information will be used in data synthesis
Data synthesis	15a	Describe criteria under which study data will be quantitatively synthesised
	15b	If data are appropriate for quantitative synthesis, describe planned summary measures, methods of handling data and methods of combining data from studies, including any planned exploration of consistency (such as I^2, Kendall's τ)
	15c	Describe any proposed additional analyses (such as sensitivity or subgroup analyses, meta-regression)
	15d	If quantitative synthesis is not appropriate, describe the type of summary planned
Meta-bias(es)	16	Specify any planned assessment of meta-bias(es) (such as publication bias across studies, selective reporting within studies)
Confidence in cumulative evidence	17	Describe how the strength of the body of evidence will be assessed (such as GRADE)

*** It is strongly recommended that this checklist be read in conjunction with the PRISMA-P Explanation and Elaboration (cite when available) for important clarification on the items. Amendments to a review protocol should be tracked and dated. The copyright for PRISMA-P (including checklist) is held by the PRISMA-P Group and is distributed under a Creative Commons Attribution Licence 4.0.**

From: Shamseer L, Moher D, Clarke M, Ghersi D, Liberati A, Petticrew M, Shekelle P, Stewart L, PRISMA-P Group. Preferred reporting items for systematic review and meta-analysis protocols (PRISMA-P) 2015: elaboration and explanation. BMJ. 2015 Jan 2;349(jan02 1):g7647.

For peer review only

EMBASE via OVID

1. child*.ab,ti.
2. exp child/
3. primary school.ab,ti.
4. youth*.ab,ti.
5. kindergar#en.ab,ti.
6. kid*.ab,ti.
7. pupil*.ab,ti.
8. juvenile*.ab,ti.
9. exp juvenile/
10. young people*.ab,ti.
11. 1 or 2 or 3 or 4 or 5 or 6 or 7 or 8 or 9 or 10
12. (actigraph* or actimet* or actograp* or actomet* or acceleromet*).ab,ti.
13. motor activity.ab,ti.
14. exp motor activity/
15. Fitbit.ab,ti.
16. ((electronic or remote or wearable or fitness or activity) adj3 (track* or monitor* or wearable* or device* or technolo*)).ab,ti.
17. step count*.ab,ti.
18. 12 or 13 or 14 or 15 or 16 or 17
19. acceptability.ab,ti.
20. experience*.ab,ti.
21. perception*.ab,ti.
22. feasibility.ab,ti.
23. feedback.ab,ti.
24. design*.ab,ti.
25. usability.ab,ti.
26. practicability.ab,ti.
27. willingness.ab,ti.
28. usefulness.ab,ti.
29. engagement.ab,ti.
30. opinion*.ab,ti.
31. 19 or 20 or 21 or 22 or 23 or 24 or 25 or 26 or 27 or 28 or 29 or 30
32. 11 and 18 and 31

For peer review only

MEDLINE via OVID

1. child*.ab,ti.
2. exp child/
3. primary school.ab,ti.
4. youth*.ab,ti.
5. kindergar#en.ab,ti.
6. kid*.ab,ti.
7. pupil*.ab,ti.
8. juvenile*.ab,ti.
9. young people*.ab,ti.
10. 1 or 2 or 3 or 4 or 5 or 6 or 7 or 8 or 9
11. (actigraph* or actimet* or actograp* or actomet* or acceleromet*).ab,ti.
12. motor activity.ab,ti.
13. exp motor activity/
14. Fitbit.ab,ti.
15. ((electronic or remote or wearable or fitness or activity) adj3 (track* or monitor* or wearable* or device* or technolo*)).ab ,ti.
16. step count*.ab,ti.
17. 11 or 12 or 13 or 14 or 15 or 16
18. acceptability.ab,ti.
19. experience*.ab,ti.
20. perception*.ab,ti.
21. feasibility.ab,ti.
22. feedback.ab,ti.
23. design*.ab,ti.
24. usability.ab,ti.
25. practicability.ab,ti.
26. willingness.ab,ti.
27. usefulness.ab,ti.
28. engagement.ab,ti.
29. opinion*.ab,ti.
30. 18 or 19 or 20 or 21 or 22 or 23 or 24 or 25 or 26 or 27 or 28 or 29
31. 10 and 17 and 30

For peer review only

PsychINFO via OVID

1. child*.ab,ti.
2. primary school.ab,ti.
3. youth*.ab,ti.
4. kindergar#en.ab,ti.
5. kid*.ab,ti.
6. pupil*.ab,ti.
7. juvenile*.ab,ti.
8. young people*.ab,ti.
9. 1 or 2 or 3 or 4 or 5 or 6 or 7 or 8
10. (actigraph* or actimet* or actograp* or actomet* or acceleromet*).ab,ti.
11. motor activity.ab,ti.
12. Fitbit.ab,ti.
13. ((electronic or remote or wearable or fitness or activity) adj3 (track* or monitor* or wearable* or device* or technolo*)).ab ,ti.
14. step count*.ab,ti.
15. 10 or 11 or 12 or 13 or 14
16. acceptability.ab,ti.
17. experience*.ab,ti.
18. perception*.ab,ti.
19. feasibility.ab,ti.
20. feedback.ab,ti.
21. design*.ab,ti.
22. usability.ab,ti.
23. willingness.ab,ti.
24. usefulness.ab,ti.
25. engagement.ab,ti.
26. opinion*.ab,ti.
27. 16 or 17 or 18 or 19 or 20 or 21 or 22 or 23 or 24 or 25 or 26
28. 9 and 15 and 27

For peer review only

Social Policy and practice via OVID

1. child*.ab,ti.
2. primary school.ab,ti.
3. youth*.ab,ti.
4. kid*.ab,ti.
5. pupil*.ab,ti.
6. juvenile*.ab,ti.
7. young people*.ab,ti.
8. 1 or 2 or 3 or 4 or 5 or 6 or 7
9. (actigraph* or actimet* or actograp* or actomet* or acceleromet*).ab,ti.
10. ((electronic or remote or wearable or fitness or activity) adj3 (track* or monitor* or wearable* or device* or technolo*)).ab ,ti.
11. 9 or 10
12. acceptability.ab,ti.
13. experience*.ab,ti.
14. perception*.ab,ti.
15. feasibility.ab,ti.
16. feedback.ab,ti.
17. design*.ab,ti.
18. usability.ab,ti.
19. willingness.ab,ti.
20. usefulness.ab,ti.
21. engagement.ab,ti.
22. opinion*.ab,ti.
23. 12 or 13 or 14 or 15 or 16 or 17 or 18 or 19 or 20 or 21 or 22
24. 8 and 11 and 23

For peer review only

For peer review only

ERIC via EBSCOhost

S1 - TI child* OR AB child* OR TI "primary school" OR AB "primary school" OR TI youth* OR AB youth* OR TI kindergar?en OR AB kindergar?en OR TI kid* OR AB kid* OR TI pupil* OR AB pupil* OR TI juvenile* OR AB juvenile* OR TI "young people*" OR AB "young people*"

S2 - TI ((actigraph* or actimet* or actograp* or actomet* or acceleromet*)) OR AB ((actigraph* or actimet* or actograp* or actomet* or acceleromet*)) OR TI "motor activity" OR AB "motor activity" OR TI fitbit OR AB fitbit OR TI (((electronic or remote or wearable or fitness or activity) adj3 (track* or monitor* or wearable* or device* or technolo*))) OR AB (((electronic or remote or wearable or fitness or activity) adj3 (track* or monitor* or wearable* or device* or technolo*))) OR TI "step count*" OR AB "step count*"

S3 - TI acceptability OR AB acceptability OR TI experience* OR AB experience* OR TI perception* OR AB perception* OR TI feasibility OR AB feasibility OR TI feedback OR AB feedback OR TI design* OR AB design* OR TI usability OR AB usability OR TI practicability OR AB practicability OR TI willingness OR AB willingness OR TI usefulness OR AB usefulness OR TI engagement OR AB engagement OR TI opinion* OR AB opinion*

S4 – S1 AND S2 AND S3

For peer review only

British Education Index via EBSCOhost

S1 - TI child* OR AB child* OR TI "primary school" OR AB "primary school" OR TI youth* OR AB youth* OR TI kindergar?en OR AB kindergar?en OR TI kid* OR AB kid* OR TI pupil* OR AB pupil* OR TI juvenile* OR AB juvenile* OR TI "young people*" OR AB "young people*"

S2 - TI ((actigraph* or actimet* or actograp* or actomet* or acceleromet*)) OR AB ((actigraph* or actimet* or actograp* or actomet* or acceleromet*)) OR TI "motor activity" OR AB "motor activity" OR TI fitbit OR AB fitbit OR TI (((electronic or remote or wearable or fitness or activity) adj3 (track* or monitor* or wearable* or device* or technolo*))) OR AB (((electronic or remote or wearable or fitness or activity) adj3 (track* or monitor* or wearable* or device* or technolo*))) OR TI "step count*" OR AB "step count*"

S3 - TI acceptability OR AB acceptability OR TI experience* OR AB experience* OR TI perception* OR AB perception* OR TI feasibility OR AB feasibility OR TI feedback OR AB feedback OR TI design* OR AB design* OR TI usability OR AB usability OR TI practicability OR AB practicability OR TI willingness OR AB willingness OR TI usefulness OR AB usefulness OR TI engagement OR AB engagement OR TI opinion* OR AB opinion*

S4 – S1 AND S2 AND S3

For peer review only

CINAHL via EBSCOhost

S1 - (MH "Child+") OR TI child* OR AB child* OR TI "primary school" OR AB "primary school" OR TI youth* OR AB youth* OR TI kindergar?en OR AB kindergar?en OR TI kid* OR AB kid* OR TI pupil* OR AB pupil* OR TI juvenile* OR AB juvenile* OR TI "young people*" OR AB "young people*"

S2 - (MH "Motor Activity+") OR TI ((actigraph* or actimet* or actograp* or actomet* or acceleromet*)) OR AB ((actigraph* or actimet* or actograp* or actomet* or acceleromet*)) OR TI "motor activity" OR AB "motor activity" OR TI Fitbit OR AB Fitbit OR TI (((electronic or remote or wearable or fitness or activity) adj3 (track* or monitor* or wearable* or device* or technolo*))) OR AB (((electronic or remote or wearable or fitness or activity) adj3 (track* or monitor* or wearable* or device* or technolo*))) OR TI "step count*" OR AB "step count*"

S3 - TI acceptability OR AB acceptability OR TI experience* OR AB experience* OR TI perception* OR AB perception* OR TI feasibility OR AB feasibility OR TI feedback OR AB feedback OR TI design* OR AB design* OR TI usability OR AB usability OR TI practicability OR AB practicability OR TI willingness OR AB willingness OR TI usefulness OR AB usefulness OR TI engagement OR AB engagement OR TI opinion* OR AB opinion*

S4 – S1 AND S2 AND S3

For peer review only

Full data extraction form

Data Extraction Form		Review date:
		Ref ID:
Author, year, date		
Title		
Journal/source		
Publication type, status		
Location of study		
Qualitative/quantitative		
Aims		
Design		
Number of participants		Drop out:
Information on age		
Information on sex		
Diagnosis		
Inclusion/exclusion criteria		
Device name, description (inc. mode of delivery)		
Device purpose		
Device wear location		
Protocol wear time and intensity		Actual:
Information on protocol adherence		
Information on device engagement/incentivisation		
Acceptability assessment measures		
Description of Outcomes		
Notes		

For peer review only

Examining the acceptability of actigraphic devices in children using qualitative and quantitative approaches: protocol for a systematic review and meta-analysis

Journal:	*BMJ Open*
Manuscript ID	bmjopen-2022-070597.R2
Article Type:	Protocol
Date Submitted by the Author:	24-Jan-2023
Complete List of Authors:	Morris, Anna; King's College London Institute of Psychiatry Psychology and Neuroscience, Child and Adolescent Psychiatry<br>Telesia, Laurence; King's College London Institute of Psychiatry Psychology and Neuroscience, ; South London and Maudsley Mental Health NHS Trust,<br>Wickersham, Alice; King's College London, Institute of Psychiatry, Psychology and Neuroscience;<br>Epstein, Sophie; South London and Maudsley NHS Foundation Trust, NIHR Maudsley Biomedical Research Centre<br>Matcham, Faith; University of Sussex, School of Psychology<br>Sonuga-Barke, Edmund; King's College London,<br>Downs, Johnny; Kings College London, Institute of Psychiatry, Psychology and Neuroscience
**Primary Subject Heading**:	Mental health
Secondary Subject Heading:	Paediatrics, Qualitative research, Research methods
Keywords:	Child & adolescent psychiatry < PSYCHIATRY, Health informatics < BIOTECHNOLOGY & BIOINFORMATICS, Community child health < PAEDIATRICS, Systematic Review

SCHOLARONE™ Manuscripts

Examining the acceptability of actigraphic devices in children using qualitative and quantitative approaches: protocol for a systematic review and meta-analysis

Morris, A. C[1]., Telesia, L[1,2]., Wickersham, A [3,4]., Epstein, S[3]., Matcham, F. A. E[5]., Sonuga-Barke, E. J. S[1]., & Downs, J. M. (2021)[1,2].

[1] King's College London, London, UK, Department of Child and Adolescent Psychiatry, Institute of Psychiatry Psychology and Neuroscience; [2] South London and Maudsley NHS Foundation Trust, London, UK; [3] King's College London, London, UK, Department of Psychological Medicine, Institute of Psychiatry Psychology and Neuroscience & NIHR South London and Maudsley Biomedical Research Centre;[4] NIHR Maudsley Biomedical Research Centre, South London and Maudsley NHS Foundation Trust, London, UK, [5] School of Psychology, University of Sussex, Falmer, UK

- anna.morris@kcl.ac.uk
- laurence.telesia@kcl.ac.uk
- alice.wickersham@kcl.ac.uk
- sophie.epstein@kcl.ac.uk
- f.matcham@sussex.ac.uk
- edmund.sonuga-barke@kcl.ac.uk
- johnny.downs@kcl.ac.uk

Correspondence: Anna Morris, Department of Child and Adolescent Psychiatry, Institute of Psychiatry, Psychology and Neuroscience, King's College London, PO Box 84, De Crespigny Park, London SE5 8AF, UK; Email: anna.morris@kcl.ac.uk

For peer review only

Abstract

Introduction: Actigraphy is commonly used to record free-living physical activity in both typically and atypically developing children. While the accuracy and reliability of actigraphy have been explored extensively, research regarding young people's opinion towards these devices is scarce. This review aims to identify and synthesise evidence relating to the acceptability of actigraphic devices in 5-11-year olds.

Methods and analysis: Database searches will be applied to Embase, MEDLINE, PsychInfo and Social Policy and Practice through the OVID interface; and Education

Resources Information Center (ERIC), British Education Index and CINAHL through the EBSCO interface from January 2018 until February 2023. Supplementary forward and backward citation and grey literature database searches, including Healthcare Management Information Consortium (HMIC) and PsycEXTRA will be conducted. Qualitative and quantitative studies, excluding review articles and meta-analyses, will be eligible, without date restrictions. Article screening and data extraction will be undertaken by two review authors and disagreements will be deferred to a third reviewer. The primary outcome, actigraphic acceptability, will derive from the narrative synthesis of the main themes identified from included qualitative literature, and pooled descriptive statistics relating to acceptability identified from quantitative literature. Subgroup analyses will determine if acceptability changes as a function of the key participant and actigraphic device factors.

Ethics and dissemination: Ethical approval is not required for this systematic review as it uses data from previously published literature. The results will be presented in a manuscript and published in a peer-review journal and will be considered alongside a separate stream of co-design research to inform the development of a novel child-worn actigraphic device.

PROSPERO Registration number: CRD42021232466

Article Summary

- Detailed study methodology and statistical analysis strategy are planned, to enable the inclusion of quantitative and qualitative literature.
- Comprehensive search terms are informed by a theoretical framework to enhance the likelihood of detecting literature relevant to acceptability.
- Literature not written in English will be excluded and may lead pertinent studies to being omitted.
- Conclusions that can be surmised from the evidence of this systematic review could be restricted by limited actigraph monitoring protocol adherence and attrition data reported in quantitative studies and the potentially low quality of identified literature.
- Narrative synthesis of qualitative evidence may be influenced by reviewer subjectivity and must be critically appraised.

Background

For peer review only - http://bmjopen.bmj.com/site/about/guidelines.xhtml

Actigraphy, an objective measure of movement, is widely considered the preferred method for activity-based monitoring in clinical and epidemiological research. [1] Modern actigraphic devices, known as actigraphs are small, inexpensive, and readily accessible rendering them ideal for inclusion in studies investigating free-living patterns of physical activity and sleep-wake cycles. [2] Actigraphs are credited for their ability to overcome important measurement biases associated with traditional subjective methods including memory, perception, and social desirability bias to produce a more detailed and accurate account of motor activity. [3] These benefits are particularly pronounced in the study of young children, where parent or teacher-reported questionnaires are often relied upon as the primary source of information, [4] and where self-reported measures tend to be inaccurate. [5, 6]

Actigraphs have been applied extensively in the remit of children's physical and mental health, especially in the context of sleep medicine, where actigraphic devices are used to record nocturnal movement patterns in children. [7] Actigraphic devices are increasingly relied upon to examine sleep patterns in childhood neurological disorders [8] and neurodevelopmental disorders. [9, 10] Deficiencies in sleep quantity or quality are also linked to the onset of various health difficulties, [11, 12] behavioural problems, and cognitive impairments. [13] Therefore, objective measures of sleep are increasingly used to support clinical assessment and management of sleep disturbances or disorders. [4] Compared to more costly data collection methods such as polysomnography which typically records sleep information for one or two nights in a laboratory, actigraphy can be used in naturalistic environments for extended periods, [14] making it easier to collect data from children who might find it difficult to sleep normally in unfamiliar surroundings. [15, 16]

Considerable efforts have also been made to deploy actigraphs to assess physical activity in children, including obesity, [17, 18] and chronic conditions such as cerebral palsy. [19] Particular attention is also being paid to the potential role of actigraphy in the assessment and diagnosis of Attention Deficit Hyperactivity Disorder (ADHD), [20] a common childhood disorder characterised by the persistent presence of three core symptoms, namely impulsivity, inattention, and hyperactivity. [21, 22] Within clinical practice, diagnosis of ADHD is usually based upon a clinical assessment of the individual, supplemented by collateral history and reports from parents/carers and teachers. [21, 23, 24, 25] However, the clinical utility of these measures has been questioned, as the main features of the disorder are rarely observed directly or systematically measured by members of the clinical decision-making team. [26] In contrast, actigraphy provides the means to objectively monitor physical activity, a well-established means of determining the severity of hyperactive symptoms, [27] across a variety of different contexts.

For peer review only - http://bmjopen.bmj.com/site/about/guidelines.xhtml

There is a considerable body of evidence focusing on the accuracy of actigraphic outcomes in the context of physical activity and sleep, [28, 29, 30] however, the question of acceptability has often been overlooked. Engagement with medical devices is heavily dependent on aesthetic preferences [31] and is potentially even more pertinent for children, who may have unique physical feature requirements compared to adults. [32] Children with neurodevelopmental disorders may also experience particular tactile sensitivities associated with the actigraphic device or its placement, [33] and stigma associated with visible markers of medical investigation. [34] Therefore, it is essential to design actigraphs that incorporate the needs and preferences of children to ensure sustained engagement with these products, particularly when asking children to wear them continuously over prolonged periods in-home and school-based environments.

Aims

This review aims to examine the literature pertaining to the acceptability of body-worn actigraphic devices for children aged 5-11 years old. Whilst this review may discover overlap between features considered acceptable by both typically and atypically developing children, differences between these groups will be highlighted if identified. If available, we aim also to include feedback on acceptability from parents/caregivers of young children who may provide insightful feedback about the acceptability of these devices. As such, the results of this review may have several practical applications, both in typically and atypically developing children. For example, by offering information to clinical teams on the types of actigraphic devices that are likely to be well tolerated by children under their care or guiding the development of future study protocols to include child-accepted actigraphs.

Further, we aim to use the findings from this review alongside data gathered from concurrent co-design research, to inform the design of a bespoke actigraphic device primarily indented to monitor physical activity within this age group.

Methods and analysis

This protocol has been prospectively recorded in the PROSPERO international database. We used the Preferred Reporting Items for Systematic Review and Meta-Analysis protocols (PRISMA-P; online supplementary file 1) checklist when writing our report. [35]

For peer review only - http://bmjopen.bmj.com/site/about/guidelines.xhtml

Eligibility criteria

Population

We are principally interested in actigraphic acceptability among primary school-aged children. In the UK, this represents children aged 5-11, an age range that typically encompasses most primary school-aged children worldwide. Therefore, studies will be included in this review if they involve children (where at least 50% fall within the 5-11 year age range or where the average age falls within this age range) in the design, development, or evaluation of an actigraphic device. Additionally, parents and caregivers of young children often act as the 'gatekeeper' responsible for enabling or restricting access to or time spent interacting with wearable technologies, [36, 37] therefore, studies including parents or caregiver/s of children aged between 5-11 years old will also be included.

Exposure and outcome

Studies will be eligible for this review if they investigate the development or use of an actigraph and report a corresponding measure of acceptability. In this review, acceptability will be defined according to the Theoretical Framework of Acceptability (TFA), [38] including participants' affective (e.g. feelings), cognitive (e.g. perceptions) or behavioural (e.g. attrition rates) response towards the actigraph. Included studies may use actigraphs for any purpose, as the primary focus of the study or as part of a multifaceted tool. Participants must be required to wear an actigraphic device in a community setting to be included in this review. Studies will be excluded if they required the wearable device to be worn in only a lab-based setting.

Study design

Qualitative studies, specifically co-design, focus groups and interview methodologies will be included as well as quantitative studies including feasibility, pilot, observational cohort, or intervention studies. Reviews, meta-analyses, and protocols will be excluded, as will commentaries, opinion pieces, editorials, magazine articles, and technical or internal reports. As patient-centred design and acceptability literature is an emerging field we will extend inclusion to published conference abstracts if they provide sufficient information to demonstrate that they meet the inclusion criteria. Likewise, letters that describe the methods and results of otherwise unpublished research may also be considered for

For peer review only - http://bmjopen.bmj.com/site/about/guidelines.xhtml

inclusion. Searches will be conducted from January 2018 until the February 2023 as this timeframe will capture the recent rapid expansion of wearable technology but is also likely to include publications which use older devices that are still suitable for use and potentially pertinent to current-day children. Studies can be published or unpublished but must be written in English. See table 1 for more detailed inclusion and exclusion criteria.

Table 1. Inclusion/exclusion table for studies exploring actigraphic devices in children.

	Inclusion criteria	Exclusion criteria
Publication status	Studies written in English<br>Published or unpublished studies	Studies written in other languages<br>Commentaries, opinion pieces, editorials, magazine articles and technical or internal reports
Study design	Qualitative or quantitative studies	Reviews or meta-analyses
Device	Studies in which a wearable activity tracker to measure motor activity was worn by a child within the target age group	Studies which do not employ any wearable devices<br>Studies using wearable devices which measure parameters other than motor activity (e.g., balance, number of steps, proportion of time sitting etc.)<br>Studies in which wearable activity trackers were discussed, but not worn
Age	Studies where all participants are aged between 5-11 years, as indicated by age range, or reported school year	Studies where all participants are outside the target age of 5-11 years

For peer review only

	Studies reporting a measure of central tendency (e.g., median, mean) which falls within the range of 5-11 years<br>Studies that present data in a way that allows extraction for a subgroup aged between 5-11 years<br>Studies that satisfy the above age criteria for the child wearing the device, but feedback is provided by a caregiver or other relevant stakeholder	Studies where it is not possible to extract data specific for participants aged between 5-11 years
Setting	Studies with protocols which involves the wear of an activity tracker in a community setting, regardless of whether the device is also worn in a non-community setting	Studies which take place entirely in a non-community setting (e.g., laboratory, university or hospital setting)
Acceptability	Studies which present a qualitative or quantitative measure of acceptability<br>Measures can include focus group or interview feedback, proportions of participants declining to wear the device or proportion and extent of deviation from the study's wear protocol	Studies which do not contain any direct or indirect measure of acceptability

Information sources and search strategy

The electronic databases Embase, MEDLINE, PsychInfo and Social Policy and Practice through the OVID interface; and Education Resources Information Center (ERIC), British

For peer review only

Education Index and CINAHL through the EBSCO interface will be searched using a combination of free text and thesaurus terms.

The search terms will derive from three key concepts, children (population), actigraphy (exposure) and acceptability (outcome). Search terms and advanced search techniques will be utilised, including truncation, wild card and adjacency operators, which will be tailored for each database as required. We will also review the reference list of included articles and related existing review articles (backward citation searching), and articles which have referenced included studies (forward citation searching). Both forward and backward citation searching will be conducted in Web of Science Core Collection, and if a citation is unavailable in this source, Google Scholar will be queried.

Additional grey literature searches will be conducted within PsycEXTRA, a complementary database to PsychINFO and the Healthcare Management Information Consortium (HMIC), comprising records from the Department of Health (DoH) and the King's Fund. Supplementary searches will be carried out using the same search strategy described above for the primary electronic databases. In addition, consultation with experts will be conducted and authors of studies included in the review will be contacted to inquire about any known studies matching the inclusion criteria. Full search strategies for the database are provided as follows; Embase (online supplementary file 2), MEDLINE (online supplementary file 3), PsychInfo (online supplementary file 4), Social Policy and Practice (online supplementary file 5), ERIC (online supplementary file 6), British Education Index (online supplementary file 7) and CINAHL (online supplementary file 8).

Data management

The search output will be downloaded into EndNote version 20 reference management software. Duplicates will automatically be detected and then removed by the reviewers. The remaining references will be exported to a Microsoft Excel document and manually screened for undetected duplicates.

Study selection process

Two reviewers (AM and LT) will each independently screen a 10% sample of the titles and abstracts generated by the search strategy. Interrater reliability for this sample will be determined using Cohen's kappa. [39] If the interrater reliability is considered "substantial" or "almost perfect", based on a kappa statistic greater than 0.60 [40] each review author will

For peer review only - http://bmjopen.bmj.com/site/about/guidelines.xhtml

independently review 50% of the remaining abstracts, with the option to jointly discuss unclear or otherwise challenging abstracts. If the strength of agreement for the 10% overlap sample is lower, both authors will review all remaining abstracts. Any disagreements between the reviewers regarding suitability for inclusion will be rectified by joint decision and, if the dispute continues, a third reviewer (JD) will decide. Full-text screening will be performed for studies deemed eligible or of unclear eligibility, again by the same two independent reviewers and a third reviewer where disagreement regarding inclusion persists. AM and LT will perform backward and forward citation searches to identify any other relevant literature. To establish agreement, this process will be conducted by both review authors (AM & LT) for 10% of the included reference lists, and the remainder evenly distributed between the authors for independent screening. Further study inclusion will be determined using the previously described consensus approach. All article exclusions will be recorded and will be justified for any articles removed at the full-text stage.

Data items and extraction process

Each paper selected for inclusion will be reviewed and data contained within the paper will be extracted and recorded using a data extraction form adapted from the Cochrane Review Group Data Extraction Form (online supplementary file 9). Both review authors (AM & LT) will perform data extraction and any disagreements regarding this procedure will be discussed and settled by a third reviewer (JD) if required. Before starting, extraction consistency will be examined by piloting the form with 10% of eligible studies. Any disagreements arising from this process will be resolved through discussion between review authors (AM and LT), and a third reviewer will decide on unresolved disagreements (JD). If needed, supplementary information will be requested directly from study authors to finalise inconsistencies and complete extraction.

For both qualitative and quantitative papers, extracted data will include - study characteristics (e.g. aims, design, sample size and analysis), information source (e.g. journal article or conference abstract), country and date of origin, title, and publication status. Participant descriptive data, including age, gender, and mental/physical health status will be recorded. Where provided, details regarding the actigraphic device will also be recorded, including device name, purpose (e.g. sleep, obesity, ADHD symptom tracking), features (e.g. material, design and functionality), body-worn location and mode of delivery (e.g. on its own or part of a multi-faceted tool).

All text relating to relevant qualitative findings will be collected from the results section of included papers, to record themes and sub-themes pertaining to child-worn actigraph

For peer review only - http://bmjopen.bmj.com/site/about/guidelines.xhtml

acceptability and engagement. Particular attention will be paid to any design or contextual factors that hinder or promote actigraphic device use in the target population. Where available, this will include direct participant quotes to afford greater data granularity at the data synthesis stage. Additionally, descriptions of the research methods used to assess acceptability (e.g. structured questionnaire with space to report free text, structured or semi-structured interview or focus groups) and the information reporting source (e.g. child, caregiver, or teacher) will be collected.

Where provided, actigraph monitoring protocol adherence data will be extracted - that is the proportion of time in the given measurement period that the actigraphic device was actually worn for the duration specified in the study methods, according to self-report e.g. diary entry, or activity monitoring device data. We will also extract absolute numbers for the duration and intensity of prescribed and actual wear time, to investigate adherence according to protocol differences. Attrition rates and protocol deviations, that is the percentage of consented participants who stop wearing the study device or wore the device in a different way than directed within the observed timeframe, as well as any comments for this behaviour will be captured. Where studies also use questionnaires, scales, or other methods to ascertain acceptability/useability, the name and content of the method, as well as participants' responses will be recorded. Information on factors which could influence participant device engagement will also be extracted. This could include but is not limited to, frequency of contact with the research team, explicit reminders to wear the device, for example, phone calls from the research team or automated text or email reminders and incentivisation. As this review focuses on the acceptability rather than the effectiveness of actigraphs, data relating to changes in behavioural outcomes following the use of an actigraphic device (e.g. improved sleep or increased physical activity) will not be collected.

If a study is found which includes at least 50% of the children aged 5-11 years as well as a minority outside of this age group, we will aim to extract data related only to the age group of interest. If it is not possible to do so, this will be highlighted in the finalised report.

Quality assessment

The relevant Critical Appraisal Skills Programme (CASP) UK [41] checklists will be used to assess the quality of included articles according to study design. Quality assessment for each paper included in the review will be independently conducted by two review authors (AM and LT), and any appraisal discrepancies will be resolved by a third reviewer (JD). Studies will not be excluded based on methodological quality, as the primary aim of this review is to identify literature about end users' experiences interacting with actigraphs.

For peer review only - http://bmjopen.bmj.com/site/about/guidelines.xhtml

Rather, the appraisal will be used to comment on the general quality of available literature regarding the acceptability of body-worn actigraphic devices in children.

Data synthesis and analysis

This review will utilise a systematic narrative synthesis approach. [42] The characteristics and key results for all included studies will first be presented in a table and elaborated on in the main body of the text. Summarised data will include, study aims, the types of actigraphic devices, details about the participant group and specific constructs of acceptability that were assessed.

For qualitative studies, the main themes, data collection method and analysis method (e.g. thematic, grounded theory or content analysis) will be presented. As outlined by Levitt [43], to meta-analyse the qualitative studies, data relevant to the review question will be selected from the included studies to form "meaning units" which will be compared with those from other studies to develop common themes and higher-order categories. Depending on the number and type of papers identified by the search, it may be more appropriate to simply summarise the findings at this stage. However, to allow meaningful comparisons, where possible the results will also be presented by subgroups, that is, according to device types or specific participant factors to better surmise any aesthetic requirements that are shared between or are unique to certain populations. Potential subgroups include, but will not be limited to, whether the children of interest are typically or atypically developing or have been diagnosed with a mental or physical health condition. Similarly, as the eligibility criteria span a period of considerable developmental change, the patterns of acceptability by age will be explored, as well as gender and ethnicity.

Descriptive statistics relating to acceptability will be summarised for quantitative studies, including percentages, frequencies, and measures of central tendency. If multiple studies are included that overlap sufficiently in methodology and outcome variables, random effects meta-analytical procedures will be used. For example, if possible, a pooled estimate of proportional wear time will be produced accompanied by a 95% confidence interval (CI) using the Stata command Metaprop. The same procedure will be applied where two or more studies employ the same acceptability questionnaires or other outcome measures. For example, if compliance data is presented as a binary outcome, such as a frequency count of participants deviating from prescribed wear protocol or proportion failing to achieve a minimum wear-time, the relative risk for that outcome will be calculated and meta-analytic methods for dichotomous outcomes will be considered, Statistical heterogeneity will be reported using the I^2 statistic. If ten or more studies are identified for inclusion within a meta-

For peer review only - http://bmjopen.bmj.com/site/about/guidelines.xhtml

analysis, techniques will be used to assess publication bias where appropriate, including Egger's regression test and funnel plots. Where possible, results stratified by actigraphic device type will be determined, and if the data permits, subgroup analyses will be reported according to the participant variables described for the qualitative subgroup analyses strategy.

Patient and public involvement

No patients were involved in the conception or development of this project.

Discussion

This systematic review seeks to identify and summarise the existing literature relating to actigraphic device acceptability in young children and highlight particular features that make actigraphic products more or less palatable in childhood. To our knowledge, this is the first synthesis to focus on child and parental acceptance of actigraph use in free-living settings for this age group, despite the potential impact of acceptability on wear time. [30, 32]

Our intention to include studies which focus on both the subjective, experiential reports from children and caregivers, as well as objective markers of wear time and attrition will allow a more comprehensive understanding of the role particular features within actigraphs play. For example, if qualitative reports consistently raise a feature as relevant, it may be possible to use a quantitative measure to corroborate this by comparing studies using devices with that feature to those without. Conversely, this approach may contradict the qualitative reports, thus identifying areas for further exploration and future research.

We hope that, by including studies with a range of prescribed wear times as well as focusing primarily on community settings, our findings will have an impact on real-world research and clinical applications. For example, some features may promote compliance in the short term only, whereas another feature is associated with better prolonged wear. This knowledge, along with an understanding of the preferences of specific subgroups, would allow clinicians and researchers to select the optimal device for their needs, and support designers to develop new products targeted to specific applications. This is timely given the rise of commercially available actigraphs purposefully developed for this age group. [36]

Furthermore, by collating and meta-analysing wear time and attrition data from several studies we hope to set a benchmark, against which new devices may be compared. Such a

For peer review only - http://bmjopen.bmj.com/site/about/guidelines.xhtml

benchmark could be helpful to device designers, particularly those wishing to evaluate prototypes against existing models in an iterative design process.

To strengthen this review, we have used a theoretical framework, the TFA to develop the search strategy, as well as use broad search terms to maximise the chances of identifying papers relevant to the study question. A possible limitation is that the process of deducing themes across varied qualitative studies has been criticised owing to the potential interference of reviewer subjectivity on the study findings. [44] However, we have brought together a team of reviewers from multi-disciplinary backgrounds to minimise any undue influence from any one reviewer's judgment. Our decision to exclude non-English written literature may limit the number of papers identified but is necessary due to resource constraints.

Ethics and dissemination

This systematic review will use existing data and therefore does not require ethical approval. Findings from the review will be published in a peer-review journal and used to inform the development of a new actigraphic device.

Footnotes:

Contributors: AM (corresponding author) and LT coconceived and developed the review protocol, defined the search strategy, drafted the initial, revised, and finalised manuscripts. AM and LT will perform data extraction and analysis and interpret the review findings. AW and SE provided critical revisions to the study design and manuscript. JD, ESB and FM coconceived the review, supported the study design and amendments to the manuscript.

Funding statement:

This review is funded by the National Institute for Health Research (NIHR) Clinician Science Fellowship award (CS-2018-18-ST2-014). LT is funded by the NHIR as an Academic Clinical Fellow. AW is in receipt of a PhD studentship funded by the NIHR Biomedical Research Centre (BRC) at South London and Maudsley NHS Foundation Trust and Kings College London (NIHR-INF-0690). SE is funded by a Medical Research Council (MRC) Clinical Research Training Fellowship (MR/T001437/1) and previously received salary support from an MQ Data Science Award and from the Psychiatry Research Trust. JD is funded by the Clinician Science Fellowship award (CS-2018-18-ST2-014) and received additional funding from an MRC Clinical Research Training Fellowship (MR/L017105/1) and Psychiatry

For peer review only - http://bmjopen.bmj.com/site/about/guidelines.xhtml

Research Trust Peggy Pollak Research Fellowship in Developmental Psychiatry. ESB is funded by the BRC, MRC (MR/T046864/1), Economic and Social Research Council (ESRC; ES/S004467/1), UK Research and Innovation's (UKRI) ESRC (ES/V016393/1) and NIHR Programme Grants for Applied Research (PGfAR; RP-PG-0618-20003).

Disclaimer: This paper represents independent research [part] funded by the NIHR Biomedical Research Centre at South London and Maudsley NHS Foundation Trust and King's College London. The funding body has not been involved in the design of the study, the collection or analysis of data, or the interpretation of data.

Competing interests statement: None declared.

References:

1. Migueles, J. H., Cadenas-Sanchez, C., Ekelund, U., Delisle Nyström, C., Mora-Gonzalez, J., Löf, M., Labayen, I., Ruiz, J. R., & Ortega, F. B. (2017). Accelerometer data collection and processing criteria to assess physical activity and other outcomes: A systematic review and practical considerations. *Sports Medicine*, 47(9),1821-1845. doi: 10.1007/s40279-017-0716-0.

2. Martin, J. L., & Hakim, A. D. (2011). Wrist actigraphy. *Chest*, 139(6), 1514-1527. doi: 10.1378/chest.10-1872.

3. Arvidsson, D., Fridolfsson, J., & Borjesson, M. (2019). Measurement of physical activity in clinical practice using accelerometers. *Journal of Internal Medicine*, 286, 137–153. doi:10.1111/joim. 12908

4. Mazza, S., Bastuji, H., & Rey, A. E. (2020). Objective and subjective assessments of sleep in children: Comparison of actigraphy, sleep diary completed by children and parents' estimation. *Frontiers in psychiatry*, 11, 495. doi: 10.3389/fpsyt.2020.00495

5. Adamo, K. B., Prince, S. A., Tricco, A. C., Connor-Gorber, S., & Tremblay, M. A. (2009). Comparison of indirect versus direct measures for assessing physical activity in the pediatric population: A systematic review, *International Journal of Pediatric Obesity*, 4:1, 2-27. doi: 10.1080/17477160802315010

6. Chinapaw, M. J. M., Mokkink, L. B., van Poppel, M. N. M., van Mechelen, W., & Terwee, C. B. (2010). Physical activity questionnaires for youth: A systematic review

For peer review only - http://bmjopen.bmj.com/site/about/guidelines.xhtml

of measurement properties. *Sports Medicine*, 40 (7), 539-563. doi:10.2165/11530770-000000000-00000.

7. Meltzer, L. J., Montgomery-Downs, H. E., Insana, S. P., & Walsh, C. M. (2012). Use of actigraphy for assessment in pediatric sleep research. *Sleep Medicine Reviews*, 16(5):463-75. doi: 10.1016/j.smrv.2011.10.002.

8. Tsai, S. Y., Lee, W. T., Jen, S. F., Lee, C., & Weng, W. C. (2018). Sleep and behavior problems in children with epilepsy. *Journal of Pediatric Health Care*, 18, 30123-8. doi: 10.1016/j.pedhc.2018.07.004

9. Merbler, A. M., Byiers, B. J., Garcia, J. J., Feyma, T. J., & Symons, F. J. (2018). The feasibility of using actigraphy to characterize sleep in Rett syndrome. *Journal of Neurodevelopmental Disorders*, 10(1), 8. doi: 10.1186/s11689-018-9227-z.

10. De Crescenzo, F., Licchelli, S., Ciabattini, M., Menghini, D., Armando, M., Alfieri, P., Mazzone, L., Pontrelli, G., Livadiotti, S., Foti, F., Quested, D., & Vicari, S. (2016). The use of actigraphy in the monitoring of sleep and activity in ADHD: A meta-analysis. *Sleep medicine reviews*, *26*, 9–20. doi: 10.1007/s12402-013-0122-x

11. Cappuccio, F. P., Taggart, F. M., Kandala, N. B., Currie, A., Peile, E., Stranges, S., & Miller, M. A. (2008). Meta-analysis of short sleep duration and obesity in children and adults. *Sleep*, 31, 619–26. doi:10.1093/sleep/31.5.619

12. Smaldone, A., Honig, J. C., & Byrne, M. W. (2007). Sleepless in America: Inadequate sleep and relationships to health and well-being of our nation's children. *Pediatrics*, 119, S29–37. doi:10.1542/peds.2006-2089F.

13. Maski, K. P., & Kothare, S. V. (2013). Sleep deprivation and neurobehavioral functioning in children. *International Journal of Psychophysiology*, 89, 259–64. doi: 10.1016/j.ijpsycho.2013.06.019.

14. Verhulst, S. L., Schrauwen, N., De Backer, W. A., & Desager, K. N. (2006). First night effect for polysomnographic data in children and adolescents with suspected sleep disordered breathing. *Archives of Disease in Childhood*, 91, 233–7. doi:10.1136/adc.2005.085365.

For peer review only - http://bmjopen.bmj.com/site/about/guidelines.xhtml

15. Sadeh, A., Alster, J., Urbach, D., & Lavie, P. (1989). Actigraphically based automatic bedtime sleep-wake scoring: Validity and clinical applications. *Journal of Ambulatory Monitoring*, 2, 209–216.

16. Sadeh, A., Lavie, P., Scher, A., Tirosh, E., & Epstein, R. (1991). Actigraphic home-monitoring sleep-disturbed and control infants and young children: A new method for pediatric assessment of sleep-wake patterns. *Pediatrics*, 87, 494–499.

17. Jiménez-Pavón, D., Kelly, J., & Reilly, J. J. (2010). Associations between objectively measured habitual physical activity and adiposity in children and adolescents: Systematic review. *International Journal of Pediatric Obesity*, 5(1), 3-18. doi: 10.3109/17477160903067601.

18. Wilks, D. C., Besson, H., Lindroos, A. K., Ekelund, U. (2011). Objectively measured physical activity and obesity prevention in children, adolescents and adults: A systematic review of prospective studies. *Obesity Reviews*, 12(5), e119-29. doi: 10.1111/j.1467-789X.2010.00775.x.

19. Keawutan, P., Bell, K., Davies, P. S., & Boyd, R. N. (2014). Systematic review of the relationship between habitual physical activity and motor capacity in children with cerebral palsy. *Research in Developmental Disabilities*, 35(6), 1301-9. doi: 10.1016/j.ridd.2014.03.028.

20. Subcommittee on Attention-Deficit/Hyperactivity Disorder, Steering Committee on Quality Improvement and Management, Wolraich, M., Brown, L., Brown, R. T., DuPaul, G., Earls, M., Feldman, H. M., Ganiats, T. G., Kaplanek, B., Meyer, B., Perrin, J., Pierce, K., Reiff, M., Stein, M. T., & Visser, S. (2011). ADHD: Clinical practice guideline for the diagnosis, evaluation, and treatment of attention-deficit/hyperactivity disorder in children and adolescents. *Pediatrics*, *128*(5), 1007–1022. doi:10.1542/peds.2011-2654.

21. De Crescenzo, F., Armando, M., Mazzone, L., Ciliberto, M., Sciannamea, M., Figueroa, C., Janiri, L., Quested, D., & Vicari, S. (2014). The use of actigraphy in the monitoring of methylphenidate versus placebo in ADHD: A meta-analysis. *Attention deficit and hyperactivity disorders*, *6*(1), 49–58. doi: 10.1007/s12402-013-0122-x.

For peer review only - http://bmjopen.bmj.com/site/about/guidelines.xhtml

22. National Institute for Health & Care Excellence. (2018). *Attention Deficit Hyperactivity Disorder: Diagnosis and Management of ADHD in Children, Young People and Adults (National Clinical Practice Guideline Number 87)*. Retrieved from: https://www.nice.org.uk/guidance/ng87/resources/attention-deficit-hyperactivity-disorder-diagnosis-and-management-pdf-1837699732933

23. American Psychiatric Association. (2013). *Diagnostic and statistical manual of mental disorders (5th ed.).* Washington, D.C.: American Psychiatric Association

24. McConaughy, S. H., Ivanova, M., Antshel, K., Eiraldi, R. B., & Dumenci, L. (2009). Standardized observational assessment of Attention Deficit Hyperactivity Disorder combined and predominantly inattentive subtypes: II. Classroom observations. *School Psychology Review*, 39, 362–381.

25. Wolraich, M. L., Chan, E., Froehlich, T., Lynch, R. L., Bax, A., Redwine, S. T., Ihyembe, D., & Hagan, J. F. Jr. (2019). ADHD diagnosis and treatment guidelines: A historical perspective. *Pediatrics*, 144(4), e20191682. doi: 10.1542/peds.2019-1682.

26. Hall, C. L., Taylor, J. A., Newell, K., Baldwin, L., Sayal, K., & Hollis, C. (2016). The challenges of implementing ADHD clinical guidelines and research best evidence in routine clinical care settings: Delphi survey and mixed-methods study. *BJPsych Open*, 2(1), 25-31. doi: 10.1192/bjpo.bp.115.002386

27. Hall, C. L., Valentine, A. Z., Groom, M. J., Walker, G. M., Sayal, K., Daley, D., Hollis, C. (2016). The clinical utility of the continuous performance test and objective measures of activity for diagnosing and monitoring ADHD in children: A systematic review. *European Child and Adolescent Psychiatry*, 25(7), 677-99. doi: 10.1007/s00787-015-0798-x.

28. Kam, H. J., Lee, K., Cho, S. M., Shin, Y. M., Park, R. W. (2011). High-resolution actigraphic analysis of ADHD: A wide range of movement variability observation in three school courses - a pilot study. *Healthcare Informatics Research*, 17(1), 29-37. doi: 10.4258/hir.2011.17.1.29.

29. Evenson, K. R., Goto, M. M., & Furberg, R. D. (2015). Systematic review of the validity and reliability of consumer-wearable activity trackers. *The international*

For peer review only - http://bmjopen.bmj.com/site/about/guidelines.xhtml

journal of behavioral nutrition and physical activity, 12, 159. doi: https://doi.org/10.1186/s12966-015-0314-1

30. Scott, H., Lack, L., & Lovato, N. (2020). A systematic review of the accuracy of sleep wearable devices for estimating sleep onset. *Sleep medicine reviews*, 49, 101227. doi: 10.1016/j.smrv.2019.101227.

31. O'Connor, S., Hanlon, P., O'Donnell, C. A., Garcia, S., Glanville, J., & Mair, F. S. (2016). Understanding factors affecting patient and public engagement and recruitment to digital health interventions: A systematic review of qualitative studies. *BMC Medical Information Decision Making*. 16(1), 120. doi: 10.1186/s12911-016-0359-3

32. Geljins, A. C., Killelea, B., Vitale, M., Mankad, V., & Moskowitz. A. (2006). Appendix C. The dynamics of pediatric device innovation: putting evidence in context. Safe Medical Devices for Children. Washington, DC: *The National Academies Press*. doi: 17226/11313

33. Fawkes, D. B., Malow, B. A., Weiss, S. K., Reynolds, A. M., Loh, A., Adkins, K. W., Wofford, D. D., Wyatt, A. D., Goldman, S. E. (2014). Conducting actigraphy research in children with neurodevelopmental disorders-a practical approach. *Behavioral Sleep Medicine*,12, 116. doi: 10.1080/15402002.2013.854245.

34. Mueller, A. K., Fuermaier, A. B., Koerts, J., & Tucha, L. (2012). Stigma in attention deficit hyperactivity disorder. *Attention Deficit Hyperactivity Disorder*. 4(3),101-14. doi: 10.1007/s12402-012-0085-3.

35. Moher, D., Shamseer, L., Clarke, M., Ghersi, D., Liberati, A., Petticrew, M., et al. (2015). Preferred reporting items for systematic review and meta-analysis protocols (PRISMA-P) 2015 statement. *Systematic Reviews*, 4(1): 1.doi: 10.1186/2046-4053-4-1

36. Mackintosh, K. A., Chappel, S. E., Salmon, J., Timperio, A., Ball, K., Brown, H., Macfarlane S, Ridgers ND. (2019). Parental perspectives of a wearable activity

For peer review only - http://bmjopen.bmj.com/site/about/guidelines.xhtml

tracker for children younger than 13 years: Acceptability and usability study. *JMIR mHealth uHealth*, 7(11), e13858. doi: 10.2196/13858

37. Dias, P., Brito, R., Ribbens, W., Daniela, L. (2016). The role of parents in the engagement of young children with digital technologies: Exploring tensions between rights of access and protection, from “Gatekeepers” to “Scaffolders”. *Global Studies of childhood*, 6, 414-427. doi: 10.1177/2043610616676024

38. Sekhon, M., Cartwright, M. & Francis, J. J. (2017). Acceptability of healthcare interventions: An overview of reviews and development of a theoretical framework. *BMC Health Services Research,* 17, 88. doi:10.1186/s12913-017-2031-8

39. Marston, L. (2010). *Introductory Statistics for Health and Nursing Using SPSS*. Sage Publications, Ltd.

40. Landis, J. R., & Koch, G. G. (1977). The measurement of observer agreement for categorical data. *Biometrics*, *33*(1), 159–174.

41. Critical Appraisal Skills Programme UK. (n.d.). *CASP checklists.* Retrieved from https://casp-uk.net/casp-tools-checklists/

42. Ryan, R., & Cochrane Consumers and Communication Review Group. (2013). ‘*Cochrane Consumers and Communication Review Group: data synthesis and analysis’*. Retrieved from: http://cccrg.cochrane.org.

43. Levitt H. M. (2018). How to conduct a qualitative meta-analysis: Tailoring methods to enhance methodological integrity. *Psychotherapy research : journal of the Society for Psychotherapy Research*, *28*(3), 367–378. doi:10.1080/10503307.2018.1447708

44. Thomas, J., & Harden, A. (2008). Methods for the thematic synthesis of qualitative research in systematic reviews. *BMC Medical Research Methodologies*, 1, 8-45. doi: 10.1186/1471-2288-8-45.

For peer review only - http://bmjopen.bmj.com/site/about/guidelines.xhtml

PRISMA-P (Preferred Reporting Items for Systematic review and Meta-Analysis Protocols) 2015 checklist: recommended items to address in a systematic review protocol*

Section and topic	Item No	Checklist item
**ADMINISTRATIVE INFORMATION**		
Title:		
Identification	1a	Identify the report as a protocol of a systematic review
Update	1b	If the protocol is for an update of a previous systematic review, identify as such
Registration	2	If registered, provide the name of the registry (such as PROSPERO) and registration number
Authors:		
Contact	3a	Provide name, institutional affiliation, e-mail address of all protocol authors; provide physical mailing address of corresponding author
Contributions	3b	Describe contributions of protocol authors and identify the guarantor of the review
Amendments	4	If the protocol represents an amendment of a previously completed or published protocol, identify as such and list changes; otherwise, state plan for documenting important protocol amendments
Support:		
Sources	5a	Indicate sources of financial or other support for the review
Sponsor	5b	Provide name for the review funder and/or sponsor
Role of sponsor or funder	5c	Describe roles of funder(s), sponsor(s), and/or institution(s), if any, in developing the protocol
**INTRODUCTION**		
Rationale	6	Describe the rationale for the review in the context of what is already known
Objectives	7	Provide an explicit statement of the question(s) the review will address with reference to participants, interventions, comparators, and outcomes (PICO)
**METHODS**		
Eligibility criteria	8	Specify the study characteristics (such as PICO, study design, setting, time frame) and report characteristics (such as years considered, language, publication status) to be used as criteria for eligibility for the review
Information sources	9	Describe all intended information sources (such as electronic databases, contact with study authors, trial registers or other grey literature sources) with planned dates of coverage
Search strategy	10	Present draft of search strategy to be used for at least one electronic database, including planned limits, such that it could be repeated
Study records:		
Data management	11a	Describe the mechanism(s) that will be used to manage records and data throughout the review

For peer review only

Selection process	11b	State the process that will be used for selecting studies (such as two independent reviewers) through each phase of the review (that is, screening, eligibility and inclusion in meta-analysis)
Data collection process	11c	Describe planned method of extracting data from reports (such as piloting forms, done independently, in duplicate), any processes for obtaining and confirming data from investigators
Data items	12	List and define all variables for which data will be sought (such as PICO items, funding sources), any pre-planned data assumptions and simplifications
Outcomes and prioritization	13	List and define all outcomes for which data will be sought, including prioritization of main and additional outcomes, with rationale
Risk of bias in individual studies	14	Describe anticipated methods for assessing risk of bias of individual studies, including whether this will be done at the outcome or study level, or both; state how this information will be used in data synthesis
Data synthesis	15a	Describe criteria under which study data will be quantitatively synthesised
	15b	If data are appropriate for quantitative synthesis, describe planned summary measures, methods of handling data and methods of combining data from studies, including any planned exploration of consistency (such as I^2, Kendall's τ)
	15c	Describe any proposed additional analyses (such as sensitivity or subgroup analyses, meta-regression)
	15d	If quantitative synthesis is not appropriate, describe the type of summary planned
Meta-bias(es)	16	Specify any planned assessment of meta-bias(es) (such as publication bias across studies, selective reporting within studies)
Confidence in cumulative evidence	17	Describe how the strength of the body of evidence will be assessed (such as GRADE)

*** It is strongly recommended that this checklist be read in conjunction with the PRISMA-P Explanation and Elaboration (cite when available) for important clarification on the items. Amendments to a review protocol should be tracked and dated. The copyright for PRISMA-P (including checklist) is held by the PRISMA-P Group and is distributed under a Creative Commons Attribution Licence 4.0.**

From: Shamseer L, Moher D, Clarke M, Ghersi D, Liberati A, Petticrew M, Shekelle P, Stewart L, PRISMA-P Group. Preferred reporting items for systematic review and meta-analysis protocols (PRISMA-P) 2015: elaboration and explanation. BMJ. 2015 Jan 2;349(jan02 1):g7647.

For peer review only

EMBASE via OVID

1. child*.ab,ti.
2. exp child/
3. primary school.ab,ti.
4. youth*.ab,ti.
5. kindergar#en.ab,ti.
6. kid*.ab,ti.
7. pupil*.ab,ti.
8. juvenile*.ab,ti.
9. exp juvenile/
10. young people*.ab,ti.
11. 1 or 2 or 3 or 4 or 5 or 6 or 7 or 8 or 9 or 10
12. (actigraph* or actimet* or actograp* or actomet* or acceleromet*).ab,ti.
13. motor activity.ab,ti.
14. exp motor activity/
15. Fitbit.ab,ti.
16. ((electronic or remote or wearable or fitness or activity) adj3 (track* or monitor* or wearable* or device* or technolo*)).ab,ti.
17. step count*.ab,ti.
18. 12 or 13 or 14 or 15 or 16 or 17
19. acceptability.ab,ti.
20. experience*.ab,ti.
21. perception*.ab,ti.
22. feasibility.ab,ti.
23. feedback.ab,ti.
24. design*.ab,ti.
25. usability.ab,ti.
26. practicability.ab,ti.
27. willingness.ab,ti.
28. usefulness.ab,ti.
29. engagement.ab,ti.
30. opinion*.ab,ti.
31. 19 or 20 or 21 or 22 or 23 or 24 or 25 or 26 or 27 or 28 or 29 or 30
32. 11 and 18 and 31

For peer review only

MEDLINE via OVID

1. child*.ab,ti.
2. exp child/
3. primary school.ab,ti.
4. youth*.ab,ti.
5. kindergar#en.ab,ti.
6. kid*.ab,ti.
7. pupil*.ab,ti.
8. juvenile*.ab,ti.
9. young people*.ab,ti.
10. 1 or 2 or 3 or 4 or 5 or 6 or 7 or 8 or 9
11. (actigraph* or actimet* or actograp* or actomet* or acceleromet*).ab,ti.
12. motor activity.ab,ti.
13. exp motor activity/
14. Fitbit.ab,ti.
15. ((electronic or remote or wearable or fitness or activity) adj3 (track* or monitor* or wearable* or device* or technolo*)).ab ,ti.
16. step count*.ab,ti.
17. 11 or 12 or 13 or 14 or 15 or 16
18. acceptability.ab,ti.
19. experience*.ab,ti.
20. perception*.ab,ti.
21. feasibility.ab,ti.
22. feedback.ab,ti.
23. design*.ab,ti.
24. usability.ab,ti.
25. practicability.ab,ti.
26. willingness.ab,ti.
27. usefulness.ab,ti.
28. engagement.ab,ti.
29. opinion*.ab,ti.
30. 18 or 19 or 20 or 21 or 22 or 23 or 24 or 25 or 26 or 27 or 28 or 29
31. 10 and 17 and 30

For peer review only

PsychINFO via OVID

1. child*.ab,ti.
2. primary school.ab,ti.
3. youth*.ab,ti.
4. kindergar#en.ab,ti.
5. kid*.ab,ti.
6. pupil*.ab,ti.
7. juvenile*.ab,ti.
8. young people*.ab,ti.
9. 1 or 2 or 3 or 4 or 5 or 6 or 7 or 8
10. (actigraph* or actimet* or actograp* or actomet* or acceleromet*).ab,ti.
11. motor activity.ab,ti.
12. Fitbit.ab,ti.
13. ((electronic or remote or wearable or fitness or activity) adj3 (track* or monitor* or wearable* or device* or technolo*)).ab ,ti.
14. step count*.ab,ti.
15. 10 or 11 or 12 or 13 or 14
16. acceptability.ab,ti.
17. experience*.ab,ti.
18. perception*.ab,ti.
19. feasibility.ab,ti.
20. feedback.ab,ti.
21. design*.ab,ti.
22. usability.ab,ti.
23. willingness.ab,ti.
24. usefulness.ab,ti.
25. engagement.ab,ti.
26. opinion*.ab,ti.
27. 16 or 17 or 18 or 19 or 20 or 21 or 22 or 23 or 24 or 25 or 26
28. 9 and 15 and 27

For peer review only

Social Policy and practice via OVID

1. child*.ab,ti.
2. primary school.ab,ti.
3. youth*.ab,ti.
4. kid*.ab,ti.
5. pupil*.ab,ti.
6. juvenile*.ab,ti.
7. young people*.ab,ti.
8. 1 or 2 or 3 or 4 or 5 or 6 or 7
9. (actigraph* or actimet* or actograp* or actomet* or acceleromet*).ab,ti.
10. ((electronic or remote or wearable or fitness or activity) adj3 (track* or monitor* or wearable* or device* or technolo*)).ab ,ti.
11. 9 or 10
12. acceptability.ab,ti.
13. experience*.ab,ti.
14. perception*.ab,ti.
15. feasibility.ab,ti.
16. feedback.ab,ti.
17. design*.ab,ti.
18. usability.ab,ti.
19. willingness.ab,ti.
20. usefulness.ab,ti.
21. engagement.ab,ti.
22. opinion*.ab,ti.
23. 12 or 13 or 14 or 15 or 16 or 17 or 18 or 19 or 20 or 21 or 22
24. 8 and 11 and 23

For peer review only

For peer review only

ERIC via EBSCOhost

S1 - TI child* OR AB child* OR TI "primary school" OR AB "primary school" OR TI youth* OR AB youth* OR TI kindergar?en OR AB kindergar?en OR TI kid* OR AB kid* OR TI pupil* OR AB pupil* OR TI juvenile* OR AB juvenile* OR TI "young people*" OR AB "young people*"

S2 - TI ((actigraph* or actimet* or actograp* or actomet* or acceleromet*)) OR AB ((actigraph* or actimet* or actograp* or actomet* or acceleromet*)) OR TI "motor activity" OR AB "motor activity" OR TI fitbit OR AB fitbit OR TI (((electronic or remote or wearable or fitness or activity) adj3 (track* or monitor* or wearable* or device* or technolo*))) OR AB (((electronic or remote or wearable or fitness or activity) adj3 (track* or monitor* or wearable* or device* or technolo*))) OR TI "step count*" OR AB "step count*"

S3 - TI acceptability OR AB acceptability OR TI experience* OR AB experience* OR TI perception* OR AB perception* OR TI feasibility OR AB feasibility OR TI feedback OR AB feedback OR TI design* OR AB design* OR TI usability OR AB usability OR TI practicability OR AB practicability OR TI willingness OR AB willingness OR TI usefulness OR AB usefulness OR TI engagement OR AB engagement OR TI opinion* OR AB opinion*

S4 – S1 AND S2 AND S3

For peer review only

British Education Index via EBSCOhost

S1 - TI child* OR AB child* OR TI "primary school" OR AB "primary school" OR TI youth* OR AB youth* OR TI kindergar?en OR AB kindergar?en OR TI kid* OR AB kid* OR TI pupil* OR AB pupil* OR TI juvenile* OR AB juvenile* OR TI "young people*" OR AB "young people*"

S2 - TI ((actigraph* or actimet* or actograp* or actomet* or acceleromet*)) OR AB ((actigraph* or actimet* or actograp* or actomet* or acceleromet*)) OR TI "motor activity" OR AB "motor activity" OR TI fitbit OR AB fitbit OR TI (((electronic or remote or wearable or fitness or activity) adj3 (track* or monitor* or wearable* or device* or technolo*))) OR AB (((electronic or remote or wearable or fitness or activity) adj3 (track* or monitor* or wearable* or device* or technolo*))) OR TI "step count*" OR AB "step count*"

S3 - TI acceptability OR AB acceptability OR TI experience* OR AB experience* OR TI perception* OR AB perception* OR TI feasibility OR AB feasibility OR TI feedback OR AB feedback OR TI design* OR AB design* OR TI usability OR AB usability OR TI practicability OR AB practicability OR TI willingness OR AB willingness OR TI usefulness OR AB usefulness OR TI engagement OR AB engagement OR TI opinion* OR AB opinion*

S4 – S1 AND S2 AND S3

For peer review only

CINAHL via EBSCOhost

S1 - (MH "Child+") OR TI child* OR AB child* OR TI "primary school" OR AB "primary school" OR TI youth* OR AB youth* OR TI kindergar?en OR AB kindergar?en OR TI kid* OR AB kid* OR TI pupil* OR AB pupil* OR TI juvenile* OR AB juvenile* OR TI "young people*" OR AB "young people*"

S2 - (MH "Motor Activity+") OR TI ((actigraph* or actimet* or actograp* or actomet* or acceleromet*)) OR AB ((actigraph* or actimet* or actograp* or actomet* or acceleromet*)) OR TI "motor activity" OR AB "motor activity" OR TI Fitbit OR AB Fitbit OR TI (((electronic or remote or wearable or fitness or activity) adj3 (track* or monitor* or wearable* or device* or technolo*))) OR AB (((electronic or remote or wearable or fitness or activity) adj3 (track* or monitor* or wearable* or device* or technolo*))) OR TI "step count*" OR AB "step count*"

S3 - TI acceptability OR AB acceptability OR TI experience* OR AB experience* OR TI perception* OR AB perception* OR TI feasibility OR AB feasibility OR TI feedback OR AB feedback OR TI design* OR AB design* OR TI usability OR AB usability OR TI practicability OR AB practicability OR TI willingness OR AB willingness OR TI usefulness OR AB usefulness OR TI engagement OR AB engagement OR TI opinion* OR AB opinion*

S4 – S1 AND S2 AND S3

For peer review only

Full data extraction form

Data Extraction Form		Review date:
		Ref ID:
Author, year, date		
Title		
Journal/source		
Publication type, status		
Location of study		
Qualitative/quantitative		
Aims		
Design		
Number of participants		Drop out:
Information on age		
Information on sex		
Diagnosis		
Inclusion/exclusion criteria		
Device name, description (inc. mode of delivery)		
Device purpose		
Device wear location		
Protocol wear time and intensity		Actual:
Information on protocol adherence		
Information on device engagement/incentivisation		
Acceptability assessment measures		
Description of Outcomes		
Notes		

For peer review only
